# Social robot navigation: a review and benchmarking of learning-based methods

**DOI:** 10.3389/frobt.2025.1658643

**Published:** 2025-12-11

**Authors:** Rashid Alyassi, Cesar Cadena, Robert Riener, Diego Paez-Granados

**Affiliations:** 1 Spinal Cord Injury and Artificial Intelligence Lab, D-HEST, ETH Zurich, Zürich, Switzerland; 2 Sensory-Motor Systems Lab, Institute of Robotics and Intelligent Systems, ETH Zurich, Zürich, Switzerland; 3 Digital Healthcare and Rehabilitation, Swiss Paraplegic Research, Nottwil, Switzerland; 4 Robotics Systems Lab, Institute of Robotics and Intelligent Systems, ETH Zurich, Zürich, Switzerland

**Keywords:** social navigation, human-robot interaction, reinforcement learning, robot learning, human-aware navigation, path planning

## Abstract

For autonomous mobile robots to operate effectively in human environments, navigation must extend beyond obstacle avoidance to incorporate social awareness. Safe and fluid interaction in shared spaces requires the ability to interpret human motion and adapt to social norms—an area that is being reshaped by advances in learning-based methods. This review examines recent progress in learning-based social navigation methods that deal with the complexities of human-robot coexistence. We introduce a taxonomy of navigation methods and analyze core system components, including realistic training environments and objectives that promote socially compliant behavior. We conduct a comprehensive benchmark of existing frameworks in challenging crowd scenarios, showing their advantages and shortcomings, while providing critical insights into the architectural choices that impact performance. We find that many learning-based approaches outperform model-based methods in realistic coordination scenarios such as navigating doorways. A key highlight is the end-to-end models, which achieve strong performance by directly planning from raw sensor input, enabling more efficient and adaptive navigation. This review also maps current trends and outlines ongoing challenges, offering a strategic roadmap for future research. We emphasize the need for models that accurately anticipate human movement, training environments that realistically simulate crowded spaces, and evaluation methods that capture real-world complexity. Advancing these areas will help overcome current limitations and move social navigation systems closer to safe, reliable deployment in everyday environments. Additional resources are available at: https://socialnavigation.github.io.

## 1 Introduction

Social navigation enables robots to move safely and efficiently in human-shared environments while respecting social norms and prioritizing human comfort. It builds on standard collision avoidance navigation by incorporating behaviors such as maintaining social distance, interpreting social cues, and predicting human movements. As a key component of *Human-Robot Interaction (HRI)*, social navigation focuses on understanding and enhancing interactions between humans and robots in shared environments.

The importance of social navigation was recognized as early as the 1990s with pioneering robots like *RHINO* (Burgard et al., 1999) and *MINERVA* (Thrun et al., 2000), which operated in dynamic environments such as museums, requiring socially aware navigation systems to interact effectively with visitors. Since then, social navigation has gained research interest, leading to steady advancements over the past years.

Several review papers reflect the interdisciplinary nature of social navigation. Sociological and human factors are addressed by Rios-Martinez et al. (2015), who apply proxemics theory, and Thomaz et al. (2016), who review computational human-robot interaction. Perception and mapping in social contexts are discussed by Charalampous et al. (2017), while safety in human-robot interaction is analyzed by Lasota et al. (2017). Path planning and navigation are extensively reviewed by Mohanan and Salgoankar (2018), Sánchez-Ibáñez et al. (2021), and Zhou et al. (2022), although mainly for classical methods. For social navigation specifically, recent surveys cover human-aware navigation (Kruse et al., 2013), conflict prevention (Mirsky et al., 2021), visual navigation (Möller et al., 2021), evaluation (Gao and Huang, 2022; Mavrogiannis et al., 2023), and taxonomy (Singamaneni et al., 2024). Human motion prediction surveys include Rudenko et al. (2020a), Sighencea et al. (2021), and Korbmacher and Tordeux (2022), comparing data-driven and model-based approaches. However, there remains a gap for a comprehensive survey focused on learning-based social navigation approaches.

This survey advances learning-based social navigation by comprehensively reviewing recent methods and introducing a novel taxonomy that categorizes algorithms into five groups by neural network architecture and system modules, expanding on earlier works like Zhu and Zhang (2021). We examine key system components, including human detection, tracking, prediction, and crowd simulation. Furthermore, our conclusions are grounded in an experimental benchmark over state-of-the-art social navigation algorithms, featuring challenging scenarios such as corridors, doorways, and intersections—areas often overlooked in previous surveys (Mavrogiannis et al., 2023). By rigorously comparing existing methods, we identify best practices, evaluate algorithm performance on new scenarios, and highlight open challenges and future directions, providing a comprehensive guide for developing learning-based social navigation systems.

The structure of this survey is as follows: Section 1 introduces a taxonomy of the social navigation problem. Section 2 presents the proposed taxonomy of social navigation algorithms and reviews recent learning-based methods. In Section 3, we examine training processes for navigation models, including discussions on objective functions, crowd simulation, and methods for human detection, tracking, and prediction. Section 4 presents an experimental comparison to validate our analysis by evaluating multiple algorithms across various simulated scenarios. Finally, Section 5 provides a discussion of existing challenges and proposes future research directions to advance social navigation.

### 1.1 Social navigation problem

Social navigation refers to a robot’s ability to navigate environments while considering human presence, social norms, and behaviors. This field encompasses a variety of navigation tasks, broadly classified into three main categories: independent, assistive, and collaborative navigation (Singamaneni et al., 2024).

#### 1.1.1 Independent

Independent crowd-aware navigation involves robots autonomously reaching goals in human-populated environments while minimizing disruption, as seen with service robots in malls or airports integrating into pedestrian flows (Yao et al., 2019). This includes systems designed for joining moving groups (Truong and Ngo, 2017) or avoiding stationary crowds (Tsoi et al., 2022). Independent navigation is the most widely studied and versatile form of social navigation.

#### 1.1.2 Assistive

Assistive navigation tasks involve robots directly supporting humans, such as follower robots in airports (Gupta et al., 2016), shopping assistants (Chen Y. et al., 2017), interactive guides (Burgard et al., 1999; Thrun et al., 2000), and systems aiding visually impaired individuals (Chuang et al., 2018), or accompanying people and groups (Ferrer et al., 2017; Repiso et al., 2020). Some tasks include proactively offering guidance (Kato et al., 2015). These tasks require detecting, following, and interpreting human cues for safe and seamless assistance.

#### 1.1.3 Collaborative

Collaborative navigation features robots and humans working together on shared tasks, either physically or through shared control. In industry, cobots assist on assembly lines (Matheson et al., 2019), while human mobility robots use shared-control systems, model-based (Gonon et al., 2021) or learning-based (Zhang et al., 2023) to integrate human input and dynamically adapt to real-time feedback.

In addition to task-based classification, social navigation can be categorized by communication strategies, focusing on how robots interact with humans through signals. For a more in-depth discussion on taxonomy, refer to Singamaneni et al. (2024) and Mirsky et al. (2021).

This review focuses on independent (crowd-aware) navigation due to its broad applicability. Its core principles can be extended to assistive and collaborative tasks, making it a more general foundation for various social navigation tasks.

## 2 Social navigation algorithms

**FIGURE 1:**
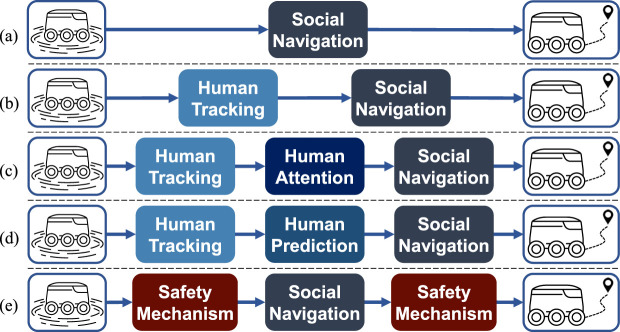
Taxonomy of Social Navigation Based on Architecture and Components outlined in Sections 2.1–2.5: **(a)** End-to-End, **(b)** Human Position-based, **(c)** Human Attention-based, **(d)** Human Prediction-based, **(e)** Safety-aware.

This section explores a range of learning-based social navigation algorithms designed for crowd-aware robot navigation. These methods function as local planners and require integration with a global planner for long-term navigation. Learning-based social navigation enables robots to navigate safely around humans through trial and error or imitation. The algorithms are categorized based on their neural network architecture and the specific modules they require, such as human detection, tracking, and prediction. This classification organizes social navigation strategies into five main categories, ranging from simpler end-to-end models to sophisticated multi-policy and prediction-based methods (see Figure 1). Furthermore, within each category, we outline several subtopics that describe common methodological themes. These themes are prevalent in certain categories but are not necessarily unique to them.

### 2.1 End-to-end navigation

End-to-end reinforcement learning (RL) (see Table 1) has proven highly effective across domains like robot navigation and autonomous driving (Bojarski, 2016). In end-to-end RL, the policy maps observations directly to actions, bypassing predefined intermediary steps and enabling complex behavior learning through trial and error. Typically, the robot’s state $s_{r}=\left[z,v_{x},v_{y},d_{gx},d_{gy}\right]$ , where z represents sensory inputs, and other navigation-related parameters such as velocity v and goal-relative distance $d_{g}$ .

**TABLE 1:** End-to-end social navigation algorithms.

Method	Input	Architecture	Algorithm	Output	Training sim	Real-world demo	Code	Training type
Wang et al. (2018a)	2D LiDAR	1D CNN + FC	Q-learning	Discrete (v,ω)	Custom Sim	None	No	Constant velocity (overlapping) obstacles
Hoeller et al. (2021)	RGB-D	VAE + LSTM + FC	PPO	$\left(v_{x},v_{y},\omega \right)$	Isaac Gym	Demo + Video	No	Constant velocity obstacles
CrowdMove (Fan et al., 2018)	2D LiDAR	1D CNN + FC	PPO	(v,ω)	Stage simulator	Demo + Video	No	Multi-agent
Liang et al. (2021)	RGB-D + 2D LiDAR	2D CNN + 1D CNN + FC	PPO	(v,ω)	Gazebo	Demo + Video	No	Agent-Based Crowd Sim (Narang et al., 2015)
Jin et al. (2020)	2D LiDAR	2D CNN + FC	DDPG	(v,ω)	Custom Sim	Demo + Video	No	ORCA (non-cooperative) crowd
Tai et al. (2018)	Depth image	ResNet-50 +FC + 2D CNN	GAIL + TRPO	(v,ω)	Gazebo	Demo + Video	Yes	SFM crowd sim
NavRep (Dugas et al., 2021)	2D LiDAR	LSTM + VAE + FC	World Models + PPO	$\left(v_{x},v_{y},\omega \right)$	Custom Sim	Demo + Video	Yes	ORCA + Constant Velocity + Global planning crowd
Cui et al. (2021)	2D LiDAR	LSTM + 3D CNN + FC	TD3	(v,ω)	Gazebo	Demo	Yes	Multi-agent
Han et al. (2022a)	RGB + 2D LiDAR	2D CNN + FC	PPO	(v,ω)	Stage simulator	Demo	No	Multi-agent
Choi et al. (2019)	2D LiDAR	2D CNN + 1D CNN + LSTM + FC	SAC	(v,ω)	Custom Sim	Demo + Video	No	Multi-agent
Cheng et al. (2023)	2D LiDAR + Preference	FC	Q-Learning	Discrete (v,ω)	Custom Sim	None	No	SFM crowd sim
Choi et al. (2020)	2D LiDAR	GRU + FC	SAC	(v,ω)	Custom Unity Sim	Demo + Video	No	Multi-agent
Lee et al. (2023)	2D LiDAR	1D CNN + FC	SAC + HER	(v,ω)	Custom Sim	Demo	Yes	Learning-based (Long et al., 2018) + global planning crowd sim

Q-learning is one of the earliest learning-based navigation methods, initially designed for static environments (Smart and Kaelbling, 2000; Smart and Kaelbling, 2002; Yang et al., 2004) and later extended to dynamic settings (Yen and Hickey, 2004; Costa and Gouvea, 2010; Jaradat et al., 2011). For instance, Wang et al. (Wang Y. et al., 2018) use a two-stream Q-network (Simonyan and Zisserman, 2014) that processes spatial (current LiDAR) and temporal (scan-difference) inputs to explicitly capture obstacle motion. These streams are processed and combined via fully connected layers, enabling effective detection of moving obstacles. While historically notable, end-to-end Q-learning is now rarely used in social navigation due to its difficulty in handling the continuous action spaces needed for smooth, realistic motion.

Actor-critic methods are widely used for continuous action spaces, addressing Q-learning’s limitations. Actor-critic models have been applied to both static (Tai et al., 2017; Zhang et al., 2017; Gao et al., 2020) and dynamic environments (Faust et al., 2018; Chiang et al., 2019). For instance, Hoeller et al. (2021) employ the PPO algorithm in combination with an LSTM network to train a robot to navigate a simulated environment. To train for dynamic collision avoidance, the environment is populated with both static and dynamic (constant-velocity) obstacles.

An alternative to using dynamic obstacles for collision avoidance training is multi-agent reinforcement learning (MARL). MARL often leverages the concept of *centralized learning with decentralized execution* to develop cooperative navigation policies (Zhang et al., 2021). In this setup, all agents are trained within a shared environment, with each agent aiming to reach its designated goal while avoiding collisions with others (Chen W. et al., 2019; Tan et al., 2020). It’s *decentralized* since there is no direct communication between agents; however, the training is *centralized* since agents share the same policy parameters and update their experiences collectively during training. For instance, Long et al. (Long et al., 2018) implemented a parallel PPO algorithm to train multiple agents to navigate in simulation. The policy is conditioned on relative goal information and 2D LiDAR data from the past three time steps, which is processed by a 1D CNN. This approach was later validated with real-world scenarios (Fan et al., 2018). Although agents trained through MARL efficiently learn to avoid collisions with other agents running an identical policy, the approach is often sub-optimal in social navigation contexts, since we assume that all agents exhibit similar behaviors, which may not reflect the diverse and adaptive behaviors in real social interactions.

An alternative to MARL is training navigation policies with simulated crowds. Here, simulated humans exhibit cooperative or reactive behaviors resembling real crowds, enabling agents to adapt to diverse social settings. For instance, Liang et al. (2021) uses PPO to train agents among cooperative, human-like agents that follow predefined paths and preferred velocities, adjusting their speed based on available space (Narang et al., 2015). Conversely, Jin et al. (2020) trains a DDPG-based policy in simulation with non-cooperative, ORCA-modeled humans (Van Den Berg et al., 2011), who react to obstacles and others without considering the robot’s path. The agent’s state is captured by multiple 2D LiDAR scans, decoupled from its motion and adjusted for heading differences over time, effectively highlighting dynamic obstacles independently of the robot’s motion.

#### 2.1.1 Learning from demonstration

Imitation learning (IL) enables learning an end-to-end policy directly from expert demonstrations, bypassing the need for hand-crafted rewards. While inverse reinforcement learning (IRL) infers a reward function from human demonstrations or pedestrian datasets (Kim and Pineau, 2016; Fahad et al., 2018) then learns a policy, behavioral cloning (BC) learns actions directly from demonstrations but struggles in dynamic settings due to its reliance on fixed data. More advanced IL approaches aim to overcome these limitations. One of the earliest data-driven approaches for static obstacle navigation, proposed by Pfeiffer et al. (2017), uses a goal-conditioned model with 1D CNN and pooling layers trained using BC. The model takes in 2D LiDAR readings and goal information to predict actions and is trained on demonstration data collected using the dynamic window approach (DWA) planner (Fox et al., 1997). While effective in static environments, this approach does not incorporate past observations, reducing its effectiveness in dynamic obstacle scenarios. Similarly, CANet (Long et al., 2017) applies behavioral cloning to learn a navigation policy from multi-agent data generated using ORCA planner (Van Den Berg et al., 2011). The model is an MLP trained to output a probability distribution over 61 pre-defined 2D velocity clusters, capturing a range of socially aware navigational behaviors. A value iteration network (VIN)-based planner, proposed by Liu et al. (2018), applies VIN (Tamar et al., 2016) to social navigation. VIN introduces a neural network architecture with a differentiable planning module that approximates the classical value iteration algorithm. Given a reward map and local transition model, VIN iteratively maps rewards and previous value estimates into Q-values using convolutional layers, where each channel corresponds to an action’s outcome. A channel-wise max pooling layer retrieves the maximum over actions, yielding the updated value function, which is then used by a greedy reactive policy network (e.g., softmax) to generate an action distribution. Liu et al. (2018) extend VIN by adding an MLP that combines the VIN output with the robot’s velocity to predict actions. Trained in a supervised manner on real and synthetic maps with demonstration actions derived from a reactive optimization-based planner, this approach provides a novel perspective on navigation. However, it’s limited to static environments and needs to be extended to dynamic settings with crowds. Another approach, GAIL, is used by Tai et al. (2018) to train a navigation policy. GAIL employs a generator (policy) that processes depth images to predict actions, while a discriminator distinguishes between the generator’s actions and expert demonstrations. To stabilize training, the discriminator is defined as a regression network, inspired by WGAN (Arjovsky et al., 2017), rather than a standard classifier. Initially, the policy is pre-trained with behavioral cloning on expert data and then fine-tuned using TRPO with the discriminator. The main advantage of GAIL is its use of online simulation-based training, which helps mitigate generalization issues. MuSoHu (Nguyen et al., 2023) addresses data scarcity in data-driven navigation by providing a large-scale dataset of 100 km of human navigation patterns collected with a helmet-mounted sensor suite. Applying behavioral cloning on this dataset produces a human-like path-planning policy that mitigates behavior modeling inaccuracies and shows strong real-world performance. DeepMoTIon (Hamandi et al., 2019) aims to mimic human pedestrian behavior by using imitation learning to train a navigation policy. The approach uses pedestrian datasets to simulate human-centric LiDAR data, training an LSTM-based policy through supervised learning. The model predicts the pedestrian’s future direction and velocity based on its LiDAR data and final goal. To account for variability in human behavior, it employs a Gaussian distribution for direction prediction, enabling the capture of diverse movement patterns in similar scenarios.

#### 2.1.2 Model-based RL

World models provide agents with internal representations of environment dynamics, enabling more informed, end-to-end decision-making. One prominent example is NavRep (Dugas et al., 2021), which integrates the World Model framework (Ha and Schmidhuber, 2018) with the PPO algorithm to train a policy. NavRep introduces *rings*, a novel 2D LiDAR representation that arranges data into exponentially spaced radial intervals within a polar coordinate grid, enhancing close-range resolution. Similarly, Cui et al. (2021) applies world models with the TD3 algorithm in a MARL framework, with the state represented by stacked 2D obstacle maps generated from multiple LiDAR scans.

#### 2.1.3 Enhanced perception methods

Most methods discussed so far rely on a single sensor input, which can be prone to noise and limited in accuracy. To enhance perception robustness for end-to-end systems, sensor fusion techniques are employed. For example, Liang et al. (2021) processes 2D LiDAR data using a 1D CNN and depth images using a 2D CNN, with inputs collected over three consecutive time steps, and combines the outputs through concatenation. In another approach, Han et al. (Han Y. et al., 2022) propose a fusion network that integrates RGB images and 2D LiDAR data to produce depth information. The 2D LiDAR data is first transformed into the camera’s coordinate frame, then combined with RGB data through an encoder-decoder CNN network (Ma and Karaman, 2018) to produce a depth image. The depth image is processed by a self-attention module, which prioritizes pixels based on factors such as robot type, goal position, and velocity, thus enhancing the agent’s situational awareness. Some navigation systems focus on optimizing performance with sensors that have limited fields of view. In these setups, self-supervised and supervised approaches are used to improve the agent’s situational awareness. For example, Choi et al. (2019) employ an actor-critic algorithm where the actor network uses an LSTM, while the critic receives additional information, such as a local 2D map. This approach allows the actor to rely on temporal cues, while the critic aids in evaluating action choices more accurately. Similarly, Monaci et al. (2022) introduce a method where an initial policy is trained using privileged information, such as precise human positions within the environment. This policy is subsequently distilled into a non-privileged policy that learns to approximate the privileged information through supervised learning.

#### 2.1.4 Multi-objective and hierarchical RL

Multi-objective reinforcement learning (MORL) (Roijers et al., 2013) frameworks are increasingly applied in end-to-end navigation tasks where agents must balance multiple, often conflicting, objectives. MORL allows a policy to be trained on multiple different objectives, enabling the adjustment of objective weightings, referred to as a preference vector, during deployment (Hayes et al., 2022). This flexibility is particularly beneficial in dynamic social environments, where safety, efficiency, and comfort are key yet sometimes competing. For example, Cheng et al. (2023) implement a vectorized Q-learning-based MORL algorithm to train a policy with a simulated crowd. Meanwhile, Choi et al. (2020) use the SAC MORL algorithm to train a navigation policy with a preference vector learned from human feedback, sampled through a Bayesian neural network (Blundell et al., 2015). Hierarchical reinforcement learning (HRL) divides complex tasks into manageable sub-tasks or sub-goals, allowing an agent to focus on different levels of decision-making. In HRL architectures, the high-level policy selects sub-goals, while the low-level policies execute these sub-goals through specific navigation actions. For instance, Lee et al. (2023) propose an HRL framework in which the high-level policy focuses on reaching the goal efficiently, minimizing time-to-goal. This policy generates a skill vector, which is then interpreted by the low-level policy to execute specific navigation skills, such as collision avoidance, goal-reaching, and maintaining a safe distance. Both levels of policy utilize 2D LiDAR data and goal state information. Other HRL approaches offer variations in task distribution and shared information. Zhu and Hayashibe (2022) use a high-level policy as a safety controller to halt the low-level policy if necessary, while Wang et al. (2021) implement an HRL framework in which the high-level policy shares a sub-goal with the low-level navigation policy.

#### 2.1.5 Vision-based navigation

In vision-based end-to-end navigation, RGB or RGB-D cameras provide input for agents to reach goals specified by relative position (PointGoal), target images (ImageGoal), or instructions (Vision-Language Navigation). These planners excel in visually rich settings without global maps, relying solely on relative goal information. Policies typically use CNN-RNN architectures, where CNNs process images and RNNs build an internal map (Kulhánek et al., 2019). Even *blind* agents, lacking vision but using memory-based policies, can navigate efficiently via spatial awareness and wall-following strategies (Wijmans et al., 2023). Such methods use photorealistic simulators based on real-world scans (Chang et al., 2017) and often employ discrete actions for training efficiency. Vision-based social navigation is emerging, with proximity-aware (Cancelli et al., 2023) and Falcon (Gong et al., 2024) methods using auxiliary tasks to better anticipate and navigate around pedestrians and obstacles.

#### 2.1.6 Language models in navigation

Vision-language models (VLMs) are powerful multimodal models with the ability to support navigation through reasoning, visual grounding, and contextual understanding. Early work on vision-language navigation (VLN) (Anderson et al., 2018a) introduced text-based high-level planning, which can be extended to social navigation for local decision-making (Li et al., 2024). Beyond high-level planning, several recent hybrid methods integrate VLMs directly into the social navigation pipeline. Song et al. (2024) use a VLM to select high-level direction and speed, which are integrated with goal and obstacle costs in a model-based planner, with weights determined through an additional VLM prompt. GSON (Luo et al., 2025) leverages VLMs to detect social groups and integrates the results into an MPC planner to generate paths that avoid them. OLiVia-Nav (Narasimhan et al., 2025) distills social context from a large VLM into lightweight encoders that provide semantic inputs to a trajectory planner, which then generates candidate motions and selects the one most aligned with captions distilled from expert demonstrations. OLiVia-Nav further incorporates lifelong learning to update its encoders with new data. Related to this, Okunevich et al. (2025) introduce an online learning approach that adapts a social module in real time, updating the social cost function during deployment. Alternatively, coding-capable large language models (LLMs) have been prompted to generate reward functions from natural language preference descriptions (Ma et al., 2023), with applications in navigation and preference alignment (Wang et al., 2024). Social-LLaVA (Payandeh et al., 2024) leverages a VLM fine-tuned for social robot navigation to directly map decisions onto a predefined set of low-level navigation primitives. Despite this progress, the slow inference and high computational demands of VLMs currently limit their use for real-time reactive social navigation. As a result, they are mostly applied as global planners, semantic encoders, or social-context modules, while their broader potential remains underexplored.

#### 2.1.7 Self-supervised learning

Beyond RL, self-supervised methods enable partial or full training of navigation policies using generated labels. For example, Hoeller et al. (2021) train a VAE to encode depth data, filter noise, and enhance sim-to-real transfer, providing informative representations for faster RL training. Yang et al. (2023) propose a bi-level framework where a neural network predicts waypoints optimized through a differentiable ESDF-based cost function; while deployment is simplified by using a spline to fit waypoints. Roth et al. (2024) further incorporate semantic costmaps, though dynamic obstacle avoidance remains unevaluated.

Overall, end-to-end navigation directly maps sensor inputs to actions and supports continuous actions, multi-agent training, model-based RL, multi-objective and hierarchical frameworks, VLMs, and self-supervised learning. However, challenges remain in ensuring safety and robustness.

### 2.2 Human position-based navigation

The challenging nature of collision avoidance in navigation has led to methods that rely on known positions and velocities of dynamic obstacles, such as humans (see Table 2). These positions are obtained through a detection and tracking module (see Section 3.3), allowing the robot to account for surrounding agents in its navigation decisions. In this setup, the human state is often represented as $s^{h}=\left[p_{x}^{h},p_{y}^{h},v_{x}^{h},v_{y}^{h}\right]$ , with the position $p^{h}$ and velocity $v^{h}$ defined in the robot frame.

**TABLE 2:** Human position-based social navigation algorithms.

Method	Input	Architecture	Algorithm	Output	Training simulator	Real-world demo	Code	Training type	Detection/ Tracking
CADRL (Chen et al., 2017b)	Human pos. and vel	FC	Deep V-learning	Sampled $\left(v_{x},v_{y}\right)$	CADRL Sim	Demo + Video	Yes	Multi-agent	LiDAR clustering (Campbell et al., 2013)
SA-CADRL (Chen et al., 2017c)	Human pos. and vel	FC	Deep V-learning	Sampled $\left(v_{x},v_{y}\right)$	CADRL Sim	Demo + Video	Yes	Multi-agent	LiDAR + RGB detection (Miller et al., 2016) LiDAR tracking (Campbell et al., 2013)
GA3C-CADRL (Everett et al., 2018)	Human pos. and vel	LSTM + FC	A3C	Sampled $\left(v_{x},v_{y}\right)$	CADRL Sim	Demo + Video	Yes	Multi-agent	LiDAR + RGB detection (Miller et al., 2016) (Liu et al., 2016), LiDAR tracking (Campbell et al., 2013)
DenseCAvoid (Sathyamoorthy et al., 2020a)	RGB-D + 2D LiDAR + Human pos. and vel	2D CNN + FC +1D CNN	PPO	(v,ω)	Custom Gazebo Sim	Demo + Video	No	Random non- cooperative crowd	YOLOv3 detection (Redmon, 2018) RobustTP prediction (Chandra et al., 2019)
Han et al. (2022b)	RVO 6D vector + Distance+ Reciprocal collision time	BiGRU + FC	PPO	$\left(\Delta v_{x},\Delta v_{y}\right)$	Custom Sim	Demo (Robot-Robot) + Video	Yes	Multi-agent	None
DRL-VO (Xie and Dames, 2023)	2D LiDAR + Human pos. and vel	2D CNN + FC	PPO	(v,ω)	Custom Gazebo Sim	Demo + Video	Yes	SFM crowd (Gloor, 2016)	YOLOv3 detection (Redmon, 2018) + MHT tracking (Yoon et al., 2018)
ILPP (Qin et al., 2021)	Human position and velocity +2D LiDAR + Global path	2D CNN + FC + Attn Block	BC	Confidence Map (guides A* planner)	None	Demo	No	Data-driven	LiDAR Detection + Kalman filter and Hungarian Algorithm
De Heuvel et al. (2022)	Human pos. and heading	FC	TD3	(v,ω)	Pybullet Simulator	Demo + Video	No	Static human	None
De Heuvel et al. (2023)	Depth image + Human pos	VAE (CNN) + LSTM + FC	TD3	(v,ω)	iGibson simulator	None	No	Static human	None
Marta et al. (2023)	2D LiDAR (labeled rays) + Human pos. and vel	FC	PPO	$\left(a_{x},a_{y}\right)$ + SFM force	Custom Unity Sim	None	No	SFM crowd	None
De Heuvel et al. (2024)	2D LiDAR + Human pos	FC	MORL-TD3	(v,ω)	iGibson sim	Demo + Video	No	Static human	None

A foundational approach in human position-based navigation is Collision Avoidance with Deep Reinforcement Learning (CADRL), introduced by Chen et al. (2017b). CADRL uses a model-based RL framework to learn a value function over the joint state space of the robot and surrounding agents. The optimal action is derived as $\pi \left(s\right)=\arg\max_{a\in \tilde{A}}V\left(\tilde{T}\left(s,a\right)\right)$ , where $\tilde{A}$ is a set of sampled actions, V is the learned value function, and $\tilde{T}$ represents the estimated transition dynamics. In CADRL, the transition dynamics model for humans is estimated using a simplified constant velocity model.

Building on CADRL, Socially Aware CADRL (SA-CADRL) (Chen et al., 2017c) incorporates social norms, such as overtaking, directly into the reward function. The value function in SA-CADRL is computed over a fixed set of agents and is trained similarly to CADRL using the multi-agent reinforcement learning (MARL) framework. Further advancements, such as GA3C-CADRL (Everett et al., 2018), extend SA-CADRL by applying the A3C algorithm and integrating an LSTM layer, enabling the policy to process an arbitrary number of agents as input, thereby increasing scalability in crowded environments. Additionally, GA3C-CADRL simplifies the reward structure by removing explicit social norms. Further research by Everett et al. (2021) explores the impact of the LSTM on this model’s performance in complex, multi-agent scenarios. While GA3C-CADRL performs well, using an LSTM to encode multiple agents may affect consistency due to LSTM’s sensitivity to input order.

A range of methods leverage the concept of velocity obstacles (VO) in state or reward functions to promote collision avoidance in navigation policies. Han R. et al. (2022) propose an RL policy that uses reciprocal velocity obstacles (RVO) (Van den Berg et al., 2008) to model agent interactions. The policy processes RVO parameters, including a 6D vector (preferred velocity and boundary velocities), distance, and reciprocal collision time for each human, using a bi-directional RNN (BiGRU). The reward function penalizes overlapping RVO areas. Some approaches, such as DRL-VO (Xie and Dames, 2023) and DenseCAvoid (Sathyamoorthy et al., 2020a), incorporate both human positions and sensor data to handle static obstacle avoidance in navigation. DRL-VO combines human positions with 2D LiDAR data, leveraging a VO-based reward function to encourage collision-free trajectories. This fusion of human position data with LiDAR enables effective static and dynamic obstacle avoidance. Similarly, DenseCAvoid uses the PPO algorithm to train a policy that fuses 2D LiDAR and RGB-D data for enhanced static obstacle detection. Building on an architecture similar to Liang et al. (2021), DenseCAvoid integrates single-step human motion predictions using RobustTP (Chandra et al., 2019), enabling the model to anticipate human movements in dynamic environments.

ILPP (Qin et al., 2021) applies imitation learning to generate a navigation confidence map that modifies the global path to incorporate collision avoidance. To produce a confidence map, the model takes LiDAR data, global path, pedestrian positions and velocities, and robot odometry. Additionally, ILPP predicts when global re-planning is necessary, especially if the expert path deviates from the global path. The model is trained using 1.3 h of a human driver operating a motorized wheelchair. To derive a path from the confidence map, the destination is set where the goal path meets the grid edge, and an A* planner finds the lowest-cost route to the destination, which is then smoothed using Gaussian filtering before being executed by a low-level controller.

#### 2.2.1 Preference-aware navigation

Approaches that incorporate human demonstrations and preferences into policy training have proven effective for aligning robot behavior with human expectations in social navigation. De Heuvel et al. (2022) use the SAC algorithm with behavioral cloning to train a policy in simulation, closely fitting human demonstration trajectories collected via a VR pointer. This work is extended in De Heuvel et al. (2023) by adding a perception pipeline that predicts future human positions. Building on this, De Heuvel et al. (2024) employ MORL-TD3 with multiple objectives, including a human demonstration distilled into a reward function using D-REX. Lastly, Marta et al. (2023) adopt a multi-objective approach to balance an expert-designed objective with a human preference objective derived from a reward model trained on pairwise human trajectory comparisons.

Overall, human position-based navigation utilizes explicit knowledge of human positions and velocities to enable safer and more socially-aware navigation policies. Techniques such as CADRL-based methods establish foundational frameworks by learning interaction-aware value functions. Moreover, incorporating human preferences and demonstrations ensures policies align closely with human expectations.

### 2.3 Human attention-based navigation

Human attention-based navigation approaches explicitly model the attention between humans within a crowd. Human Attention-based approaches have become a key component in social navigation, enabling policies that adapt to both individual and crowd dynamics, and achieving significant performance improvement (see Table 3). These methods explicitly model relationships between human features using pooling layers or graph neural networks (GNNs) to represent mutual influences. Pooling layers provide a compact, unified representation of human features, which, when combined with individual features, encodes human-human attention. In graph-based approaches, the robot and humans are nodes in the input graph, generating node embeddings that capture human-human and robot-human relationships.

**TABLE 3:** Human-human interaction-based social navigation algorithms.

Method	Input	Architecture	Algorithm	Output	Training sim	Real-world demo	Code	Detection/ Tracking
SARL (Chen et al., 2019b)	Human pos. and vel	Self-Attn + LSTM + FC	Deep V-learning	80 discrete (v,θ)	CrowdNav	Demo + Video	Yes	Depth projection detection
Liu et al. (2020a)	2D LiDAR grid/map + Human pos. and vel	Self-Attn + LSTM +2D CNN + FC	A3C	Discrete (v,ω)	Custom Sim	Demo + Video	No	MobileNetSSD + Block matching detection (Eppenberger et al., 2020)
NaviGAN (Tsai and Oh, 2020)	Human path	GAN + LSTM + Pooling + FC	GAN	${\left(p_{x},p_{y}\right)}_{1},\ldots$	None	Demo + Video	No	LiDAR + Kalman filter
DS-RNN (Liu et al., 2021)	Human position	S-RNN (LSTM/GRU) + FC	PPO	$\left(v_{x},v_{y}\right)$	CrowdNav	Demo + Video	Yes	YOLOv3 detection (Redmon, 2018) + DeepSORT tracking (Wojke et al., 2017)
GazeNav (Chen et al., 2020a)	Human pos. and vel	GCN + FC	Deep V-learning	Sampled (v,θ)	Custom Sim	None	No	None
Navistar (Wang et al., 2023a)	Human pos. and vel	Multi-Head Attn + GCN + FC	SAC	$\left(v_{x},v_{y}\right)$	CrowdNav	Lab experiment + Video	Yes	Velocity predictor (YOLO + DeepSORT) (Pramanik et al., 2021) + Distance estimator (Bertoni et al., 2021)
Liu et al. (2023a)	Human occupancy grid	GAT + 2D CNN + LSTM + FC	DQN	5 discrete $\left(v_{x},v_{y}\right)$	Custom Sim	Demo + Video	No	Yolov3 detection (Redmon, 2018) + pointcloud tracking (Liu et al., 2020b)

SARL (Chen et al., 2019b) builds on CADRL (Chen et al., 2017b) by introducing an attention and a pooling module to explicitly capture human-human attention. The attention module encodes features of each human relative to surrounding humans using a human-centered local map. In this local map, each human’s surrounding individuals are divided into grid cells concatenated with the human and robot states, then the features are passed into an MLP to produce a human embedding vector. To capture human-human attention and transform an arbitrary number of human embeddings into a fixed-size vector, SARL uses a self-attention pooling module, an attention mechanism adapted from Transformers. This attention mechanism assigns scalar weights to each human embedding vector and computes a unified output by summing the weighted embeddings across all humans. This dual-stage position-based encoding via the local map and self-attention pooling improves social navigation performance compared to methods without explicit attention encoding, though local maps offered a slight performance improvement during testing. During deployment, SARL may also be adapted to use a single-step human trajectory prediction model to estimate the next state, offering a more accurate alternative to the constant velocity model used in CADRL.

SOADRL (Liu et al., 2020a) extends SARL to a model-free RL setup, introducing a two-policy switching mechanism to address both dynamic and static obstacles. When humans are present, SOADRL combines SARL’s output with a robot-centric angular map or 2D occupancy grid for static obstacle encoding. In the absence of humans, SOADRL switches to a policy that relies solely on the map input, ensuring efficient navigation through static obstacles.

NaviGAN (Tsai and Oh, 2020) introduces a learning-based social force model (SFM) for navigation using a dual LSTM-based GAN architecture. The model’s first LSTM generates an intention force based on the robot’s goal and past state sequence, while the second LSTM generates a social force that accounts for human interactions. It uses a pooling layer similar to the one in Social-GAN (Gupta et al., 2018) to encode human history. It also incorporates a fluctuation force for randomness. The combined intention and social forces determine the robot’s future actions. A discriminator is used during training to encourage realistic behavior, distinguishing between generated actions and expert actions from a real-world pedestrian dataset. To incorporate temporal information, DS-RNN (Liu et al., 2021) uses a three-RNN architecture trained with PPO for social navigation. One RNN encodes each human’s past positions relative to the robot; another encodes the robot’s past velocities. These embeddings are combined via attention pooling (without modeling human-human attentions) and, along with the robot’s state, fed into a third RNN that outputs the policy action and value function.

#### 2.3.1 Graph neural network-based navigation

GazeNav (Chen et al., 2020a) employs a model-based RL approach with gaze-based attention that uses 2 two-layer Graph Convolutional Networks (GCNs) to define its value function. The first GCN, an attention network, treats the robot and humans as graph nodes with uniform edge weights, predicting attention weights for each connection. The second GCN is an aggregation network that uses the predicted attention weights as edge values to compute embedding vectors for each human-robot pair, which are then passed into an MLP-based value function. To train the attention network, GazeNav introduces three supervised methods: uniform weights, distance-based weights, and gaze-modulated weights. The gaze-modulated weights are obtained by tracking human gaze in a simulated environment, assigning higher attention to humans within the gaze direction. Experiments show that gaze-modulated weights outperform uniform, distance-based, and self-attention-based weights (Chen C. et al., 2019), demonstrating the benefits of incorporating human gaze data. For a more expressive representation, Navistar (Wang W. et al., 2023) uses a three-block architecture to model spatio-temporal crowd interactions. A spatial block (GCN plus multi-head attention) creates spatial embeddings; a temporal block applies multi-head attention with positional encoding for each human. A multi-modal transformer block then merges these outputs using cross-attention and self-attention to produce the final action and value outputs. In a related approach, Liu Z. et al. (2023) integrate GNNs with occupancy grids to capture spatial-temporal characteristics. At each time step, the environment is divided into a robot-centered grid and an obstacle-centered grid for each human, both processed by a CNN. The CNN outputs are then passed through an LSTM to capture temporal patterns, feeding into a Graph Attention Network (GAT) that produces interaction-aware embeddings. The control policy uses an MLP to generate action distributions from the GAT’s aggregated output.

To summarize, human attention-based navigation methods explicitly model human-human and human-robot attentions to enable socially-aware and adaptive policies. Approaches utilizing pooling layers, GNNs and RNNs, provide improved social compliance by capturing spatial and temporal relationships.

### 2.4 Human prediction-based navigation

Human Prediction-based Social Navigation (see Table 4) leverages human trajectory prediction to enable more strategic, optimal navigation in dynamic environments (see Section 3.3.2). This approach aligns with model-based RL principles, where the human prediction model serves as a dynamics model, guiding decision-making by simulating future states. To leverage this predictive capability, the navigation system should plan over a similar multi-second horizon rather than just single-step actions. Early work in this area applied techniques like Monte Carlo Tree Search (MCTS) for high-level decision-making in autonomous vehicles (Paxton et al., 2017) and optimization-based planners such as MPC for robots (Finn and Levine, 2017). One notable example is Chen et al. (2018), who use a Social-LSTM (Alahi et al., 2016) to predict human trajectories, incorporating this into an optimization-based timed elastic band (TEB) planner (Rösmann et al., 2015) with adaptive travel modes that adjust based on crowd density and movement direction.

**TABLE 4:** Human prediction-based social navigation algorithms.

Method	Input	Architecture	Algorithm	Output	Training sim	Real-world demo	Code	Detection/ Tracking	Prediction
MCTS-RNN (Eiffertet al., 2020a)	Human path	LSTM Enc-Dec + FC	MCTS (Model-based)	Path ${\left(p_{x},p_{y}\right)}_{1},\ldots$	CrowdNav	Demo + Video	Yes	None	Learned RNN
MP-RGL (Chen et al., 2020b)	Human pos. and vel.	GCN + FC	MCTS (Model-based)	Discrete paths ${\left(v_{x},v_{y}\right)}_{1},\ldots$	CrowdNav	Demo + Video	Yes	YOLO detection + EKF tracking	Learned GCN
GO-MPC (Brito et al., 2021)	Human pos. and vel.	LSTM + FC	PPO + MPC	(v,ω)	CADRL Sim	None	Yes	None	Constant velocity model
Poddar et al. (2023)	Human path	LSTM + GAN + FC	MPC (Model-based)	Discrete $\left(v_{x},v_{y}\right)$	CrowdNav	Lab experiment + Video	Yes	None	S-GAN (Gupta et al., 2018)
SARL-SGAN-KCE (Li et al., 2020)	Human pos. and vel.	GAN + LSTM + Self-Attn	Deep V-Learning	(v,ω)	CrowdNav	None	No	None	S-GAN (Gupta et al., 2018)
Liu et al. (2023b)	Human path	Multi-Head-Attn + GRU + FC	PPO	$\left(v_{x},v_{y}\right)$	CrowdNav	Demo + Video	Yes	DR-SPAAM (Jia et al., 2020)	GST (Huang et al., 2021)

#### 2.4.1 MCTS-based navigation

MCTS-RNN (Eiffert et al., 2020a) is a model-based RL navigation system that uses an LSTM encoder-decoder human prediction model as its dynamics model. The LSTM model is trained on pedestrian datasets and outputs a Gaussian distribution over future human states. Planning is conducted using MCTS with a receding horizon, performing single-step rollouts from each node to reduce runtime, which increases state uncertainty. To handle this, the reward function includes both goal proximity and prediction uncertainty. MP-RGL (Chen C. et al., 2020) integrates MCTS planning with a GCN-based human prediction model. The GCN operates on a fully connected graph comprising humans and the robot, where edge weights are computed using Gaussian similarity in the node embedding space (Wang X. et al., 2018). Planning is performed through a simplified MCTS (Oh et al., 2017), with a d -step planning horizon, leveraging value function estimates instead of explicit rollouts.

#### 2.4.2 MPC-based navigation

GO-MPC (Brito et al., 2021) is a hybrid framework that integrates RL and nonlinear MPC for navigation, where an LSTM-based RL model proposes sub-goals (as Gaussians) and the MPC computes optimal, collision-free trajectories to these sub-goals. The RL model is first supervised-trained with MPC-generated labels, then fine-tuned with PPO, aiming to maximize goal-reaching and minimize collisions. The MPC minimizes distance and control costs, enforcing constraints to avoid predicted human paths. Poddar et al. (2023) propose a hybrid approach that integrates a Social-GAN (Gupta et al., 2018) human prediction model with an MPC planner. This approach uses discrete MPC to optimize a cost function that balances goal distance, social distance, and alignment with Social-GAN predictions to encourage human-like behavior. While Social-GAN can generate multiple predictions per human, results indicate that single and multiple prediction scenarios perform comparably to simpler constant-velocity estimates.

SARL-SGAN-KCE (Li et al., 2020) combines Social-GAN predictions with the SARL model (Chen C. et al., 2019) to choose optimal single-step actions. To ensure smooth motion, the planner constrains the action space by limiting angular velocity and penalizing rapid acceleration changes. Experimental results show that a higher number of trajectory predictions per human achieves performance comparable to a lower number of predictions. Finally, Liu S. et al. (2023) propose a model-free PPO RL approach that incorporates off-the-shelf human prediction models like GST (Huang et al., 2021). Human predictions are processed with multi-head human-human attention, then through robot-human attention with the robot’s state, followed by a GRU that outputs the value and action. The reward penalizes intersecting predicted human paths, reducing collision risk despite prediction uncertainty.

In summary, human prediction-based navigation enhances decision-making by anticipating future human movements, enabling more strategic and socially compliant planning. Challenges include managing uncertainty from the robot’s impact on human behavior and the computational cost of tree-based methods like MCTS, which require repeated action sampling and forward simulation.

### 2.5 Safety-aware navigation

Considering that learning-based approaches are, in some sense, viewed as black-box methods, researchers have attempted to embed safety and functionality through purposefully designed algorithms (see Table 5). These approaches are classified as safety-aware when they introduce an additional module, training strategy, or feature primarily dedicated to safety.

**TABLE 5:** Safety-aware social navigation algorithms.

Method	Input	Architecture	Algorithm	Output	Safety mechanism	Training sim	Real-world demo	Code
Sun et al. (2019)	2D LiDAR (labeled rays)	LSTM + FC	PPO	(v,ω)	Multi-policy (VO-based switch)	Unity Custom Sim	None	No
Katyal et al. (2020)	Human path	VAE + LSTM + FC	Deep V-Learning	2D velocity	Multi-policy (uncertainty switch)	CrowdNav	None	No
Fan et al. (2020)	2D LiDAR	1D CNN + FC	PPO	(v,ω)	Multi-policy (distance switch)	Stage Sim	Lab experiment + Video	No
Linh et al. (2022)	Static and dynamic 2D LiDAR	2D CNN + FC	A3C	(v,ω)	Multi-policy (RL policy switch)	Arena-Rosnav	None	Yes
Nishimura and Yonetani (2020)	Human pos. and vel.	Self-Attn + FC	Deep V-Learning	8 discrete $\left(v_{x},v_{y}\right)$ + Clearing beep	Crowd clearing beep	Custom Sim	None	Yes
IAN (Dugas et al., 2020)	Grid map	None	MCTS	(v,ω)	Multi-policy (MCTS switch)	Custom Sim	Demo + Video	Yes
Lütjens et al. (2019)	Human pos. and vel.	LSTM + FC	MPC	11 discrete (v,θ)	Uncertainty collision prediction	Custom Sim	None	No
Sathyamoorthy et al. (2020b)	2D LiDAR + human pos	1D CNN + FC	PPO	(v,ω)	Heading Safety filter	Gazebo Sim	Demo + Video	No
XAI-N (Roth et al., 2021)	2D LiDAR	Decision Tree	PPO + VIPER	Discrete (v,ω)	Decision Tree	Gazebo Sim	Demo + Video	Yes
Jang and Ghaffari (2024)	Human pos. and vel.	None	MPC	$\left(v_{x},v_{y}\right)$	Control-Barrier Function	2D sim	None	No
CASRL (Zhou et al., 2023)	Human pos. and vel.	HGAT + MLP	TD3	$\left(v_{x},v_{y}\right)$	Multi-task	CrowdNav	Demo	No
Pfeiffer et al. (2018)	2D LiDAR	FC	CPO	(v,ω)	Collision avoidance constraint	Custom Sim	Demo	Yes
SoNIC (Yao et al., 2024)	Human pos. and vel.	GRU + MLP	PPO-Lag	$\left(v_{x},v_{y}\right)$	Constraint	CrowdNav	Demo + Video	Yes

#### 2.5.1 Multi-policy navigation

Hybrid multi-policy planning combines multiple strategies, where robots switch policies based on context and uncertainty. For example, Sun et al. (2019) switches between RL and RVO when a collision is imminent. Katyal et al. (2020) build on this with risk-averse and aggressive policies. By default, the system follows the aggressive policy but switches to the risk-averse policy in novel social scenarios, identified by an LSTM-based probabilistic pedestrian prediction module that uses goal intent prediction to generate a set of possible trajectories. The policy selector computes uncertainty from these predictions, with higher uncertainty indicating unfamiliar situations where the risk-averse policy is preferred. Extending this approach, Fan et al. (2020) develop a three-policy system with a scenario classifier to switch between a PID controller, a standard RL policy (Long et al., 2018), and a safe RL policy with clipped velocity. The classifier relies on two parameters, the *safe radius* and *risk radius*, based on the distance to nearby obstacles. When within the safe radius, the PID policy is used. In the risk radius, the RL policy takes over, and outside both, the safe policy is employed. To address more complex scenarios, Amano and Kato (Amano and Kato, 2022) add a fourth policy to this setup, a reset policy to move the robot toward a larger unoccupied space if it detects a freezing robot scenario. This extension ensures the robot can navigate out of potentially freezing situations. Furthermore, Linh et al. (2022) propose a multi-policy system with three policies, using an RL-based policy selector to choose the most appropriate policy dynamically. Policies include both learning-based (RL) and model-based (TEB) planners (Rösmann et al., 2015). The selector is trained to optimize rewards by picking the best policy for a given context, combining flexibility with performance for complex navigation tasks.

Nishimura and Yonetani (2020) introduce Learning-to-Balance (L2B), a single-policy RL system that dynamically switches between two behaviors: passive crowd avoidance or active path-clearing through audible signals. The robot action is defined by a velocity vector and a binary mode indicator, with a reward function that discourages excessive path-clearing while promoting social distancing. To simulate the impact of path-clearing sounds on human behavior during training, L2B uses a simplified version of emotional reciprocal velocity obstacles (ERVO) (Xu M. et al., 2019), which accounts for emotional reactions to perceived threats. IAN (Dugas et al., 2020) is a multi-policy navigation system that uses Monte Carlo Tree Search (MCTS) to choose among three planning policies: intend (RVO planner (Alonso-Mora et al., 2013) for reactive avoidance), say (verbal path announcement with lower speed and assumed human cooperation), and nudge (DWA planner (Fox et al., 1997) for cautious progress). MCTS evaluates paths by crowdedness, perceptivity, and permissivity, selecting the lowest-cost route and adapting plans based on each policy’s success probability. Both L2B and IAN require the robot to have a speaker and operate where its audio signals are audible.

Lütjens et al. (2019) propose a hybrid safe RL system based on discrete MPC, optimizing a cost function that accounts for estimated goal-reaching time and predicted collision probability. An ensemble of LSTMs predicts collision probabilities of motion primitives, with MC-dropout (Gal and Ghahramani, 2016) used for uncertainty estimation. The collision prediction model is trained as a binary classifier in simulation, penalizing uncertainty to encourage safe exploration. However, this approach heavily depends on collision model accuracy, and inaccuracies can lead to overly conservative behavior. Sathyamoorthy et al. (2020b) introduce Frozone, which prevents robot freezing by detecting potential freezing zones (PFZs) using pedestrian positions and velocities. A convex hull is constructed around predicted pedestrian locations, and the robot computes a deviation angle to avoid these regions. However, in confined spaces like corridors, Frozone may lead the robot toward other obstacles. XAI-N (Roth et al., 2021) leverages decision trees to create an interpretable navigation policy. XAI-N distills an RL policy (Fan et al., 2018) into a single decision tree using the VIPER method (Bastani et al., 2018), prioritizing modifiability and transparency over continuous action control. To enhance performance, the approach incorporates decision rules to address safety challenges such as freezing and oscillation, making it a more reliable option for social navigation.

Bansal et al. (2020) propose a Hamilton–Jacobi reachability-based framework that augments the human state with a belief over future intent, producing a forward reachable set that includes all likely pedestrian states for fixed time-horizon with probability above threshold λ . These sets are incorporated as time-dependent obstacles and avoided with a spline-based trajectory planner. However, in highly populated scenes, the predicted reachable sets may overlap heavily for a low threshold, effectively blocking all routes and causing the robot to freeze, and while the human model parameters can be learned from data, the overall prediction approach is model-based. Jang and Ghaffari (2024) learn social zones from pedestrian data by relating distance to line-of-sight angle, then approximate them with speed-dependent ellipses enforced through a control-barrier function within a hybrid MPC planner. The learned zones are front-biased—indicating humans prefer more space ahead—and slightly tilted, reflecting overtaking behavior. While enforcing them as hard constraints improves safety, it can be overly conservative, since people typically tolerate reduced spacing in crowded settings. CASRL (Zhou et al., 2023) frames safety in navigation as a multi-task RL problem: goal reaching and collision avoidance tasks. It extends an off-policy RL algorithm (TD3) with separate critics for each task, while the actor is updated using a conflict-averse rule that maximizes the minimum improvement across tasks. This reduces performance loss when gradient updates disagree. However, the reported simulation gains fall short and may require further tuning.

#### 2.5.2 Constrained RL

Constrained RL provides a natural framework for enforcing safety, as constraints take precedence over the reward objective when violated. For instance, Pfeiffer et al. (2018) introduce a safe RL navigation policy that defines a collision constraint, trained using constrained policy optimization (CPO) (Achiam et al., 2017), which maximizes reward while constraining the expected number of collisions. SoNIC (Yao et al., 2024) introduces a safety constraint derived from Adaptive Conformal Inference (ACI), which quantifies the uncertainty of predicted pedestrian trajectories. Similarly, Zhu et al. (2025) propose a confidence-weighted trajectory prediction model, where a Bayesian β parameter adapts the uncertainty region based on prediction errors. In their method, uncertainty is incorporated through a robust dynamical distance constraint that estimates time-to-collision, rather than relying on simple distance-based thresholds. However, both approaches employ trajectory predictors that neglect the robot’s presence, resulting in predictions where pedestrians are assumed to move independently of the robot. This leads to overly conservative robot behavior.

In conclusion, safety-aware navigation improves reliability in learning-based systems through structured mechanisms, but further work is needed to balance safety with efficiency and ensure adaptability to diverse real-world scenarios.

## 3 Navigation model training

**FIGURE 2:**
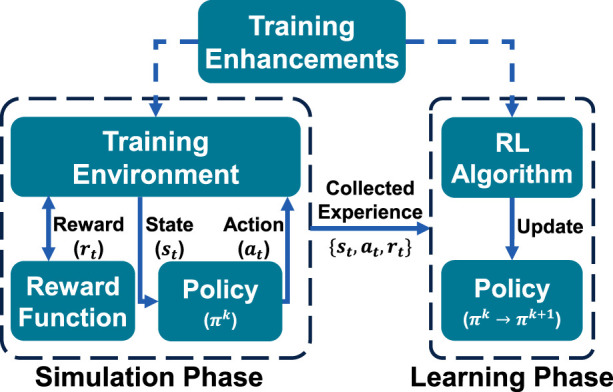
Illustration of the RL training loop, alternating between the Simulation Phase, where the navigation model (policy) interacts with the simulation environment, and the Learning Phase, where collected experience is used to improve the model through the RL algorithm.

Training social navigation policies equips robots with safe, efficient, and socially aware navigation in human environments. This section outlines key training components (see Figure 2), including the objective function, environments with static and dynamic obstacles, including realistic crowd simulation. Advanced strategies, such as pre-training, enhance training efficiency. We also examine human detection, tracking, prediction, and broader scene understanding and activity recognition, which are leveraged by navigation policies to improve performance. Finally, we cover evaluation methods for social navigation, including metrics and real-world experiments.

### 3.1 Objective function

The objective or reward function in most reinforcement learning (RL) problems is typically formulated as $\max E\left[\sum_{t=0}^{h}\gamma^{t}r_{t}\right]$ , where the goal is to maximize the expected cumulative reward. This formulation typically incorporates a discount factor γ∈[0,1] to prioritize earlier rewards over delayed ones. The reward function, denoted by r(s,a) , is often defined as a function of the current state and action but can also incorporate the subsequent state $r\left(s,a,s^{\prime }\right)$ , to capture transition dynamics. In navigation tasks, the objective is often a weighted sum of reward components:

$$r\left(s,a,s^{\prime }\right)=\underset{i}{\sum }w_{i}r_{i}\left(s,a,s^{\prime }\right)$$

where $w_{i}\in R^{+}$ are weights and r are scalar and indicator functions. The section details possible reward components, but it’s worth noting that combining multiple objectives can create local minima (Everett et al., 2021).

Many reward functions are *sparse*, providing feedback only at key milestones like reaching a goal. To improve learning, *reward shaping* introduces dense rewards, giving intermediate feedback at each timestep. While dense rewards speed up learning, they must be carefully designed to avoid suboptimal strategies.

#### 3.1.1 Goal reward

The reward function for reaching a goal state is the main component of any navigation task. It is often defined as an indicator function $\left[\|p^{r}-g^{r}\|\leq g_{\mathrm{tol}}\right]$ , which returns 1 when the Euclidean distance between the robot’s current position p and the goal position $g^{r}$ is within a specified tolerance $g_{\mathrm{tol}}$ . A dense reward formulation for goal-reaching provides feedback based on progress, calculated as $\left\|p_{t}^{r}-g\|-\|p_{t-1}^{r}-g^{r}\right\|$ , reflecting incremental advancement toward the goal (Long et al., 2018; Tan et al., 2020). Other approaches reward the agent for moving along grid cells that align with the global path (Liu Z. et al., 2023).

#### 3.1.2 Collision-avoidance reward

The reward function for collision avoidance is often defined as an indicator function, $-\left[p^{r}\in C\right]$ , which returns 1 when the robot’s position $p^{r}$ is within a collision set C , or when a collision is detected using other means. Alternative definitions penalize proximity to obstacles, such as $-\left[d_{\mathrm{obs}}|d_{\mathrm{obs}}\leq d_{\mathrm{tol}}\right]$ (Chen et al., 2017b) or $\left[\frac{d_{\mathrm{obs}}}{d_{\mathrm{tol}}}-1|d_{\mathrm{obs}}\leq d_{\mathrm{tol}}\right]$ (Cui et al., 2021) where $d_{\mathrm{obs}}$ is the minimum distance to nearby obstacles and $d_{\mathrm{tol}}$ is the tolerance distance. These formulations may apply different thresholds for static and dynamic obstacles, such as humans (Cui et al., 2021). Other approaches use step functions to gradually increase the penalty as the robot gets closer to obstacles, encouraging safer navigation.

#### 3.1.3 Efficiency reward

To encourage efficient and timely navigation, reward functions often include terms that promote higher speeds. This may take the form of a gradual step function that provides a higher reward for increased velocity (Lee and Jeong, 2023) or a negative step-cost applied at each timestep to minimize time taken to reach the goal (Wang Y. et al., 2018; Choi et al., 2019).

#### 3.1.4 Smoothness reward

For smooth trajectory generation, a negative reward proportional to the rotational velocity, −|ω| is applied for differential drive robots (Tan et al., 2020; Xie and Dames, 2023). Additionally, Hoeller et al. (2021) penalizes lateral and backward velocities by adding a negative reward proportional to their squared magnitudes, encouraging smoother and more consistent forward motion.

#### 3.1.5 Social reward

Social norms can be integrated into the reward function to promote behaviors like passing, crossing, and overtaking in socially appropriate ways (Chen et al., 2017c). This reward function is typically defined as a conditional function based on human parameters relative to the robot, including x-axis position, velocity, distance, relative heading angle, and heading angle difference. For instance, to promote overtaking from the left, the robot is rewarded when certain conditions are met: the goal distance exceeds 3, the human is positioned within $0<p_{x}<3$ , and $0<p_{y}<1$ , the robot’s velocity surpasses the human’s, and their heading angle difference is under π/4 .

#### 3.1.6 Geometric collision-avoidance reward

Model-based or geometric rewards using human position have enabled more robust navigation. For instance, *DRL-VO* (Xie and Dames, 2023) uses velocity obstacles (VOs) to model human motion, rewarding alignment with the optimal heading direction, where VOs are computed during training only. Han R. et al. (2022) incorporate VOs into both state and reward, with rewards based on joint VO area, velocity differences, and estimated minimum time to collision. Zhu et al. (2022) employ an oriented bounding capsule (OBC) model, where human velocity adds a buffer in front of the OBC, and the reward is the minimum distance to the OBC; OBC parameters are included in the robot’s state for better learning. Additionally, Samsani and Muhammad (2021) define a danger zone (DZ) as an extended sector around humans, accounting for uncertainty in position and velocity predictions.

#### 3.1.7 Human preference reward

Reinforcement Learning from Human Feedback (RLHF) provides a framework to simultaneously learn a policy and a reward function using human input (Christiano et al., 2017), with applications spanning various domains, including language models like GPT-3 (Ouyang et al., 2022). In social navigation, Wang R. et al. (2022) applies RLHF to learn a reward function based on pairwise human preferences over trajectory segments.

#### 3.1.8 Human prediction reward

For planners that utilize human trajectory predictions, a negative reward is often used to discourage the robot from intruding into human-predicted zones (Liu S. et al., 2023). Additionally, a negative reward can be defined over prediction uncertainty, as in (Eiffert et al., 2020b), where the reward is the negative square root of the determinant of the covariance matrix, $\sqrt{det\left(\Sigma \right)}$ , encouraging actions that lead to more predictable crowd behavior.

#### 3.1.9 Exploration reward

Exploration rewards are designed to encourage the robot to explore a wide range of actions or states. Action-based exploration rewards promote action diversity by maximizing the policy’s entropy (Schulman et al., 2017), while state-based exploration rewards encourage the robot to explore new areas. For instance, the intrinsic curiosity module (ICM) (Pathak et al., 2017), applied to navigation tasks (Shi H. et al., 2019; Martinez-Baselga et al., 2023) to reward the robot for discovering novel states, thereby enhancing its learning process.

#### 3.1.10 Task-specific reward

Task-specific rewards are custom-designed to achieve the requirements of a particular navigation task. For example, in social navigation with a human companion, Li et al. (2018) define a distance-based reward that penalizes the robot for straying from its companion, encouraging it to stay close and coordinate its movement with the human partner.

#### 3.1.11 Learning rewards from demonstrations

Inverse reinforcement learning (IRL) infers a reward function from expert demonstrations, either by using handcrafted state–action features (Okal and Arras, 2016; Kim and Pineau, 2016) or by learning feature representations directly with neural networks (Fahad et al., 2018). For instance, Vasquez et al. (2014) learn a reward function expressed as a weighted combination of features that capture local crowd density, relative velocities and orientations of nearby pedestrians, the robot’s own velocity, and social force interactions. More recently, methods like disturbance-based reward extrapolation (D-REX) (Brown et al., 2020) learn reward functions from suboptimal or unlabeled data. D-REX applies behavioral cloning, adds increasing ϵ -greedy noise, and trains the reward to favor less noisy trajectories, promoting desirable behavior, an approach effective for social navigation (De Heuvel et al., 2024). Similar techniques include T-REX (Brown et al., 2019) and SSRR (Chen et al., 2021).

#### 3.1.12 Learning reward weights

Various techniques have been developed to automatically determine the optimal values of each objective weight $w_{i}$ , eliminating the need for manual tuning. One such method is inverse reinforcement learning (IRL), which infers the weights $w_{i}$ by matching the robot’s behavior to expert demonstrations (Ziebart et al., 2008). Additionally, AutoRL (Chiang et al., 2019; Parker-Holder et al., 2022) employs automated hyperparameter tuning to optimize the reward weights $w_{i}$ during training to enhance task-specific performance metrics.

### 3.2 Training environment

This section reviews key components of training environments for social navigation, focusing on crowd data and physics-based simulators that replicate robot dynamics and sensory feedback to ensure realistic training conditions. Furthermore, crowd simulation libraries (see Table 6) provide controllable and realistic human behaviors that can be used to populate training environments and replicate crowd datasets.

**TABLE 6:** Crowd simulation libraries.

Library	Crowd behavior	Language
RVO2 (Van Den Berg et al., 2025)	ORCA	Python (Stüvel, 2025) or C++
UMANS (van Toll et al., 2020)	SFM, PLEdestrians, RVO ORCA, PowerLaw, Vision	C++
PySocialForce (Gao, 2025)	SFM with groups (Moussaïd et al., 2010)	Python
DeepSocialForce (Kreiss, 2021)	Deep Social Force	Python
CROMOSIM (Faure, 2025)	SFM, CA, Granular	Python
CrowdDynamics (Group, 2025)	SFM with Path planning	Python
JuPedSim (Dynamics, 2025)	SFM, Centrifugal, CFSM	Python & C++
Mesa (Masad, and Kazil, 2015)	ABM	Python
Agents.jl (Datseris et al., 2024)	ABM	Julia
Vadere (Kleinmeier et al., 2019)	CA, SFM, OSM (Seitz and Köster, 2012)	Java

#### 3.2.1 Crowd data

Crowd datasets play a critical role in advancing data-driven approaches for both crowd behavior simulation and human trajectory prediction. They provide the necessary information to model realistic crowd interactions and dynamics, as detailed in the Appendix. Additionally, these datasets support the training of human prediction methods, as explored in Section 3.3.2. Table 7 organizes these datasets by their sensory platforms, including stationary sensors, moving robots, and moving vehicles, each serving distinct purposes and applications. While long-term crowd tracking datasets such as the ATC dataset (Brščić et al., 2013) exist, they lack the scale and diversity needed to support social navigation research.

**TABLE 7:** Pedestrian datasets.

Dataset name	Collection location	Data volume	Annotations	Sensor platform
ETH (Pellegrini et al., 2009)	ETH Zurich, and Hotel in Zurich	25 min, 2 tracks	Human position	Stationary top-view camera
UCY (Lerner et al., 2007)	University campus and Zara store	16 min, 6 tracks	Human position	Stationary top-view camera
Central Station (Zhou et al., 2012)	New York train station	33 min	Human position	Stationary top-view camera
Edinburgh (Majecka, 2009)	Outdoor University campus	92,000 trajectories	Human position	Stationary top-view camera
Stanford Drone (Robicquet et al., 2016)	Stanford University campus outdoor	5 h, 60 tracks	Bounding box	Stationary drone top-view camera
VIRAT (Oh et al., 2011)	Indoor and outdoor	25 h	Bounding box	Stationary surveillance camera
Oxford Town (Benfold and Reid, 2011)	Oxford town center	5 min	Bounding box	Stationary surveillance camera
ATC (Brščić et al., 2013)	Shopping mall	92 days	Human position	Stationary top-mounted radar
Ko-PER (Strigel et al., 2014)	Crossing in Aschaffenburg, Germany	6.5 min	Human position	Stationary top-view camera and Laser
inD (Bock et al., 2020)	Crossing in Aachen, Germany	10 h	Human position	Stationary drone top-view camera
CITR and DUT (Yang et al., 2019)	University campus	13 min, 66 tracks	Human position	Stationary drone top-view camera
WILDTRACK (Chavdarova et al., 2018)	ETH Zurich, Switzerland	200 s	Human position	Stationary tilted camera
STCrowd (Cong et al., 2022)	Outdoor streets and walkways	84 tracks	Human position	Stationary vehicle with RGB and 3D LiDAR
THOR Dataset (Rudenko et al., 2020b)	Indoor large room	60 min	Human position	Stationary/moving robot with 3D LiDAR and tracking
L-CAS (Yan et al., 2017)	Indoor university building	49 min	Human position	Stationary and moving robot with 3D LiDAR
SCAND (Karnan et al., 2022)	Indoor and outdoor	8.7 h	Social interaction	Robot with RGB-D, and 3D LiDAR
JRDB (Martin-Martin et al., 2021)	Indoor and outdoor	64 min	Human position	Robot with RGB-D, and 3D LiDAR
FLOBOT (Yan et al., 2020)	Airport and supermarket in Italy/France	27 min	Human position	Autonomous robot with RGB-D, and 3D LiDAR
NCLT (Carlevaris-Bianco et al., 2016)	University campus indoor and outdoor	35 h	None	Robot with RGB-D, and 3D LiDAR
MuSoHu (Nguyen et al., 2023)	Indoor and outdoor	20 h	Social interaction	Walking human + helmet with RGB-D and 3D LiDAR
CrowdBot (Paez-Granados et al., 2021)	3 streets in Lausanne, Switzerland	200 min	Human position	Semi/Autonomous Robot with RGB-D and 3D LiDAR
HuRoN (Hirose et al., 2023)	5 office environments	75 h	Human position	Autonomous robot with RGB-D and 2D LiDAR
SiT (Bae et al., 2024)	60 scenes, indoor and outdoor, Seoul	470,000 frames	Human position	Robot with RGB-D, and 3D LiDAR
KITTI (Geiger et al., 2012)	Karlsruhe, Germany	6 h	Human position	Moving vehicle with RGB, 3D LiDAR and more
nuScences (Caesar et al., 2020)	Boston, MA and Singapore	5.5 h, 20s tracks	Human position	Moving vehicle with RGB, 3D LiDAR and more
Waymo (Ettinger et al., 2021)	San Francisco, Phoenix, and more	570 h, 20s tracks	Human position	Moving vehicle with RGB, 3D LiDAR and more
BDD100K (Yu et al., 2020)	New York, Berkeley, and more	1100 h, 40s tracks	Human position	Moving vehicle with RGB, 3D LiDAR and more
A2D2 (Geyer et al., 2020)	South of Germany	40,000 frames	Human position	Moving vehicle with RGB, 3D LiDAR and more

#### 3.2.2 Simulation platform

Simulators provide a controlled virtual environment for developing and evaluating social navigation algorithms by modeling human-robot interactions and crowd behaviors. Table 8 categorizes simulators based on key attributes, such as the supported sensor types, the human model ranging from simple cylindrical shapes to detailed 3D figures, supported crowd behaviors, evaluation metrics based on implementation specifics.

**TABLE 8:** Simulation platforms.

Simulator	Description	Human behavior	Sensors	Human model	Maps	Metrics
Menge (Curtis et al., 2016)	Supports ROS (Aroor et al., 2017)	Path planning + ORCA and SFM	None	Circle	2D Maps	No
SEAN (Tsoi et al., 2022; Tsoi et al., 2020)	Based on Unity game engine	SFM and ORCA	RGB-D	3D humanoid	3D maps	Navigation and Social
SEAN-EP (Tsoi et al., 2021)	Build on SEAN simulator For human feedback collection	SFM and ORCA	RGB-D	3D humanoid	3D maps	Navigation and Social
UnrealCV (Qiu et al., 2017)	Build on Unreal Engine 4	None	RGB	3D humanoid	3D maps	No
MORSE (Echeverria et al., 2011)	Based on Blender engine	None	RGB-D and 3D LiDAR	3D humanoid	3D maps	No
Gazebo (Koenig and Howard, 2004)	ROS compatible simulator	None	RGB-D and 3D LiDAR	3D humanoid	3D maps	No
Isaac Sim (Makoviychuk et al., 2021; Mittal et al., 2023)	Based on PhysX engine	None	RGB-D and 3D LiDAR	3D Rigid human (Isaac GYM)	None	No
Webots Sim (Michel, 2004)	Based on OpenGL engine	None	RGB-D and 3D LiDAR	3D humanoid	3D maps	No
AI2-THOR (Kolve et al., 2017)	Based on Unity Engine Only supports LoCoBot	None	RGB-D	None	3D maps	No
Habitat 2.0 (Szot et al., 2021)	Based Habitat Sim	None	RGB-D	None	3D Scans	Navigation
Habitat 3.0 (Puig et al., 2023)	Based Habitat Sim	Path Planning	RGB-D	3D humanoid	3D Scans	Navigation and Social
HabiCrowd (Vuong et al., 2023)	Based Habitat Sim	UPL (Karamouzas et al., 2014)	RGB-D	3D humanoid	3D Scans	Navigation and Social
SAPIEN (Xiang et al., 2020)	Based on PhysX engine	None	RGB-D	None	3D Scans	No
CrowdBot Sim (Grzeskowiak et al., 2021)	Based on Unity Engine	UMANS (van Toll et al., 2020)	RGB-D and 3D LiDAR	3D humanoid	3D maps	Navigation and Social
iGibson (Li et al., 2021)	Based on PyBullet	ORCA	RGB-D and 3D LiDAR	3D Rigid human	3D Scans	No
CrowdNav (Chen et al., 2019b)	2D openspace simulator	SFM and ORCA	None	Circle	None	No
Arena-Rosnav (Kästner et al., 2021)	Uses Gazebo, Unity and Flatland	SFM and ORCA	RGB-D and 3D LiDAR	Circle or 3D humanoid	2D and 3D maps	Navigation and Social
nav-gym (Lee et al., 2023)	Use CrowdNav with Flatland	ORCA	2D LiDAR	Circle or 3D humanoid	2D maps	No
gym-collision -avoidance (Everett et al., 2018)	Supports multiple robots and humans	ORCA	None	Circle	None	No
navrep (Dugas et al., 2021)	Multiple social simulators	Global planner + ORCA	2D LiDAR	Circle or human with moving legs	2D maps	No
gym ped sim (Tai et al., 2018)	A plugin for Gazebo	SFM	RGB-D and 3D LiDAR	3D humanoid	3D maps	No
Social Gym (Sprague et al., 2023)	A multi-agent 2D simulator	RL planner	None	Circle	2D maps	Navigation and Social
RDS Sim (Gonon et al., 2021)	RDS planner evaluation code	ORCA	None	Circle	None	Navigation and Social

Many simulators share a common emphasis on creating realistic environments. For instance, Habitat (Szot et al., 2021) and Gibson (Li et al., 2021), widely used in embodied AI research, render highly detailed indoor spaces using real 3D scans. However, these environments are typically limited to smaller areas like apartments or offices, making them less suitable for large-scale crowd simulations. Additionally, several simulators, such as Isaac Sim (Makoviychuk et al., 2021), prioritize achieving high FPS, which is crucial for training performance. Simulators also vary in the complexity of crowd behaviors, with some supporting basic movement patterns and others providing sophisticated, behavior-rich models that more accurately capture crowd dynamics, such as NavRep (Dugas et al., 2021). However, current simulators remain limited, as an efficient RL-supported crowd simulation with diverse scenarios is still missing, which we aim to address with our benchmark.

### 3.3 Human detection, tracking, and prediction

In social navigation, a robot often relies on human detection, tracking, or prediction for better social awareness and generalizability. *Human Detection* provides the robot-centric human positions, which are essential for position-based planners (see Section 2.2). *Human Tracking* estimates human positions and velocities over time, supporting planners requiring human speeds or trajectories. *Human Prediction* utilizes tracking data to forecast future human movements, which are utilized by prediction-based planners (see Section 2.4).

#### 3.3.1 Human detection and tracking

Human detection methods are typically tailored to specific sensors, including 2D LiDAR, 3D LiDAR, RGB, and RGB-D sensors. Tracking enhances detection by assigning unique identifiers to individuals and addressing challenges such as sensor occlusions, which is essential for reliable multi-object tracking (MOT). While human detection provides the robot-centric positions of each detected person, tracking maintains a history of these positions over time, enabling the estimation of their velocities.

##### 3.3.1.1 Human detection

RGB-based human detection leverages general object detection techniques, which can be broadly categorized into classical and deep learning approaches. Classical methods such as histogram of oriented gradients (HOG) (Dalal and Triggs, 2005) and deformable part model (DPM) (Felzenszwalb et al., 2008), often struggle with accuracy and robustness in complex or dynamic environments. Deep learning-based methods, on the other hand, are divided into one-stage and two-stage approaches. Two-stage or *coarse-to-fine* methods, like Faster R-CNN (Ren et al., 2016) and FPN (Lin et al., 2017), typically offer higher accuracy by refining proposals. While one-stage detectors, such as YOLO (Redmon, 2016), SSD (Liu et al., 2016), and DETR (Carion et al., 2020), prioritize speed, making them ideal for real-time applications in social navigation. For further details, refer to Zou et al. (2023). The output of RGB-based object detection provides a bounding box in the image plane, which requires conversion to robot-centered coordinates for accurate spatial positioning. To estimate 3D pose parameters from 2D detections, some methods, such as Multi-fusion (Xu and Chen, 2018) and ROI-10D (Manhardt et al., 2019), incorporate depth estimation modules to approximate distance. Meanwhile, techniques like Deep3DBox (Mousavian et al., 2017), MonoGRnet (Qin et al., 2019), and Hu et al. (2019) apply geometric reasoning techniques for 3D localization based on 2D information.

Early methods for 2D LiDAR-based human detection relied on hand-crafted features, identifying humans by detecting both legs within a segment (Arras et al., 2007) or by tracking individual legs over time (Leigh et al., 2015). The first deep learning-based detector, *DROW* (Beyer et al., 2016), was subsequently enhanced by incorporating temporal information to improve tracking consistency (Beyer et al., 2018). Building upon *DROW*, *DR-SPAAM* (Jia et al., 2020) introduced faster processing capabilities for handling long-term temporal data. Additionally, Dequaire et al. (2018) employed an occupancy grid-based approach combined with an RNN to capture temporal patterns effectively. Current 3D LiDAR detection approaches are categorized into Bird’s Eye View (BEV) methods, point-based methods, voxel-based methods, multi-view methods, and range-view-based methods. *BEV* methods provide fast, top-down 2D projections of the environment, making them popular for quick processing tasks in robotics. Examples include PIXOR (Yang et al., 2018a) and HDNet (Yang et al., 2018b). However, they often miss critical vertical details essential for detecting objects like pedestrians. *Point-based* methods directly process raw point cloud data, offering higher accuracy. Notable examples are PointNet++ (Qi et al., 2017) and PointRCNN (Shi S. et al., 2019). However, these methods are computationally intensive and less suitable for real-time applications. *Voxel-based* methods transform point clouds into 3D voxel grids, effectively balancing accuracy and computational efficiency by reducing processing loads while preserving essential details. Notable examples include VoxelNet (Zhou and Tuzel, 2018) and SECOND (Yan et al., 2018). *Multi-view* methods, such as MV3D (Chen X. et al., 2017) and SE-SSD (Zheng et al., 2021), combine multiple point cloud representations to leverage their respective advantages and enhance detection performance. *Range-view-based* methods convert LiDAR data into 2D range images, preserving vertical details and achieving high processing speeds, making them well-suited for applications like social navigation. Approaches include RangeNet++ (Milioto et al., 2019) and RSN (Sun et al., 2021). RGB-D-based human detection combines RGB data with depth information, which can also be acquired from a 3D LiDAR for sensor fusion. Techniques like PointPainting (Vora et al., 2020) fuse RGB semantic data onto LiDAR points, while PointNet (Qi et al., 2018) leverage 3D bounding frustums, focusing detection within the RGB-D space. For further details, refer to Mao J. et al. (2023).

##### 3.3.1.2 Human tracking

Human tracking involves identifying detected objects, assigning each object a unique ID, and continuously updating their location through state estimation filters, even during brief sensor occlusions. This section centers on the Tracking-by-Detection framework, which performs detection before tracking, as other tracking frameworks are less common for human tracking. Trackers vary by association metrics and tracking dimensionality. In general, MOT relies on motion prediction techniques such as Kalman filters, particle filters, or multi-hypothesis tracking (MHT) (Yoon et al., 2018), combined with application-specific association metrics (Rakai et al., 2022). For vision-based MOT, popular methods include DEEPSort (Wojke et al., 2017) which integrates deep association metrics, ByteTrack (Zhang et al., 2022) which relies on hierarchical association for accurate initial detection and faster performance, and other methods (Xu Y. et al., 2019). For 3D MOT, approaches like AB3DMOT (Weng et al., 2020), which utilizes 3D bounding boxes and Kalman filtering, and other approaches like SimpleTrack (Pang et al., 2022) and CAMO-MOT (Wang L. et al., 2023) which enhance tracking accuracy and efficiency. Fusion-based MOT combines 2D and 3D detections from multiple sensors to enhance tracking robustness. EagerMOT (Kim et al., 2021) fuses information from multiple detectors, while DeepfusionMOT (Wang X. et al., 2022) applies deep learning-based association for enhanced consistency. For further details, refer to Peng et al. (2024).

#### 3.3.2 Human trajectory prediction

Predicting human trajectories is critical for effective social navigation. Traditionally relying on knowledge-based methods, the field has shifted towards learning-based approaches, which consistently outperform traditional methods on metrics such as average displacement error (ADE) (Pellegrini et al., 2009) and final displacement error (FDE) (Alahi et al., 2016). Learning-based methods leverage crowd datasets (see Section 3.2.1) and typically employ CNN, LSTM, or GAN architectures (Korbmacher and Tordeux, 2022).

##### 3.3.2.1 CNN-based predictors

CNNs, initially designed for spatial tasks, have been adapted to sequential pedestrian prediction by representing trajectories spatially. Early approaches such as Behavior-CNN (Yi et al., 2016) encode pedestrian trajectories into displacement volumes processed by CNN layers. More recent models, such as Social-STGCNN (Mohamed et al., 2020), incorporate graph convolutions to effectively model pedestrian interactions, while scene context integration further enhances predictions (Ridel et al., 2020). Overall, CNNs efficiently process data in parallel but typically require reprocessing the full input history for each prediction, limiting their efficiency in real-time navigation.

##### 3.3.2.2 LSTM-based predictors

LSTM networks excel at capturing temporal dependencies in sequential data. Social-LSTM (Alahi et al., 2016) introduced social pooling to account for pedestrian interactions during prediction. Enhancements include integrating environmental context via semantic information (Lisotto et al., 2019) and employing attention mechanisms (Fernando et al., 2018). Graph-based methods like STGAT (Huang et al., 2019) further improve interaction modeling. Transformers have recently emerged as powerful alternatives, better capturing complex interactions and limited sensing scenarios (Huang et al., 2021). In contrast, LSTMs, despite slower batch processing, efficiently leverage hidden states for incremental, real-time predictions, making them ideal for social navigation.

##### 3.3.2.3 GAN-based predictors

GAN-based models generate diverse and realistic trajectories, addressing human behavior’s multi-modality. Influential methods include Social-GAN (Gupta et al., 2018), which combines LSTMs with GAN frameworks, and SoPhie (Sadeghian et al., 2019), which integrates social and physical context through attention modules. Recent advancements like probabilistic crowd GAN (PCGAN) (Eiffert et al., 2020b) and diffusion-based models (Gu et al., 2022; Mao W. et al., 2023) further enhance multi-modal, safety-compliant predictions. Despite the computational demand, GANs’ diverse trajectory predictions significantly contribute to robust and safe decision-making in social navigation scenarios.

### 3.4 Scene understanding and activity recognition

Scene understanding and activity recognition are perception modules that provide information beyond human detection and trajectory prediction. Scene understanding includes object detection, pose estimation, semantic segmentation, saliency prediction, affordance prediction, and captioning (Naseer et al., 2018).

Object detection and pose estimation, detailed in Section 3.3 for humans, can be generalized to other classes for broader scene understanding. Beyond object detection, 2D and 3D semantic segmentation assign semantic labels to pixels or points in images and LiDAR scans, producing detailed maps of the environment (Kirillov et al., 2023; Cen et al., 2023) with applications to navigation (Roth et al., 2024). Affordance prediction further interprets the scene by modeling possible interactions; for navigation, this is useful for identifying robot-traversable areas (Yuan et al., 2024). Saliency prediction models human visual attention by estimating focus regions in a scene (Lou et al., 2022), allowing vision models to ignore irrelevant input and prioritize informative areas. Finally, 3D dense captioning methods, such as Vote2Cap-DETR (Chen et al., 2023), extend scene classification or 2D captioning by generating multiple localized captions, offering richer scene descriptions for context-aware navigation.

In parallel, activity recognition interprets dynamic human behaviors at both the individual and group levels. At the individual level, this involves human action classification (Girdhar et al., 2017), while at the group level it includes group activity classification (Choi et al., 2009) often supported by group detection methods (Wang Q. et al., 2018; Li et al., 2022). More recently, LLM-based classifiers have been introduced for activity recognition (Qu et al., 2024; Liu et al., 2025). Current navigation approaches primarily use activity recognition to estimate proxemics (Charalampous et al., 2016; Narayanan et al., 2020), though its potential for richer context-aware decision-making remains unexplored.

Vision-language models (VLMs) (Liu H. et al., 2023) are large multimodal models with broad capabilities, including object recognition, reasoning, and contextual understanding. By jointly leveraging visual and textual inputs, they provide a natural bridge between scene understanding, activity recognition, and navigation guidance. Despite this potential, their use in social navigation remains limited, with only a few recent methods exploring VLM-based decision making (Song et al., 2024; Munje et al., 2025).

### 3.5 Training enhancement techniques

Efficient training is essential for robust social navigation policies, since large-scale RL training is often limited by computational resources. While extensive training, such as training a DD-PPO policy for 2 billion steps Wijmans et al. (2019), can boost performance, more efficient approaches exist. Task-specific techniques, such as leveraging problem symmetries by flipping path topologies (Chen et al., 2017c) can improve exploration. This section highlights general, task-agnostic methods for enhancing training efficiency and performance.

#### 3.5.1 Pre-training techniques

Pre-training techniques, such as behavioral cloning from demonstrations (Pfeiffer et al., 2018; Chen C. et al., 2019), accelerate training by providing basic navigation skills and reducing RL exploration. Self-supervised methods, like VAEs with reconstruction loss (Dugas et al., 2021; Hoeller et al., 2021), improve state representation, while transfer learning from pretrained CNNs enhances RGB input processing (Hong et al., 2021). Policy transfer from existing models is also used (Wijmans et al., 2019). These approaches improve training efficiency, convergence, and generalization.

#### 3.5.2 Auxiliary tasks

Auxiliary tasks are additional tasks or objectives incorporated during training to support learning the main task. This offers better training signal and model performance. Auxiliary tasks have been shown to improve navigation performance by training models to predict features such as depth, loop closures (Mirowski et al., 2016), and location estimation (Tongloy et al., 2017). Additional tasks include predicting immediate reward prediction and learning to control specific regions in the input image (Jaderberg et al., 2016) or predicting image segmentation (Kulhánek et al., 2019). In social navigation, auxiliary tasks are used to improve understanding of social dynamics. For instance, *Proximity-Aware* (Cancelli et al., 2023) incorporates tasks to estimate the distance and direction of surrounding humans, while *Falcon* (Gong et al., 2024) incorporates tasks for predicting the number of nearby humans, tracking their locations, and estimating their future trajectories. These tasks enable the model to acquire valuable insights into the environment’s social dynamics, leading to more efficient and informed planning.

#### 3.5.3 Curriculum learning

Curriculum learning gradually increases task difficulty during training, aiding convergence in challenging social navigation tasks. In RL, this process involves three steps: task generation, sequencing, and transfer learning (Narvekar et al., 2020). *Task generation* creates scenarios of varying difficulty by adjusting obstacles, goal distances, or map complexity, using parameter sampling or grid search. *Sequencing* organizes tasks by increasing difficulty, either at a fixed rate or adaptively based on agent performance, and may involve modifying reward functions or start/goal distributions (Riedmiller et al., 2018; Florensa et al., 2018), optimization strategies (Matiisen et al., 2019), Curriculum MDPs (Narvekar et al., 2017), or human feedback (Bengio et al., 2009). *Transfer learning* adapts agents when intermediate tasks differ in state/action spaces, rewards, or dynamics, such as transitioning from precise states to noisy sensors, or from indoor to outdoor navigation. This combination allows agents to efficiently learn complex social navigation skills.

#### 3.5.4 Teacher-student framework

The teacher-student framework enables a teacher model, often trained with privileged information, to guide a student via real-time feedback, reward shaping, or action labels. Knowledge transfer is achieved through policy distillation (Rusu et al., 2015), using labeled paths or actions from the teacher, student, or both (Czarnecki et al., 2018), allowing the student to imitate and refine its navigation policy, which can later be fine-tuned with RL. Teachers may also provide reward signals to enhance exploration (Czarnecki et al., 2019) and corrective action feedback (Ross et al., 2011). Model-based teachers like MPC are also used (Lowrey et al., 2018). Asymmetric actor-critic methods allow the critic to use privileged information to guide the actor (Pinto et al., 2017). In teacher-student curriculum learning, teachers assign progressively harder tasks and are rewarded for student improvement (Matiisen et al., 2019), while multi-teacher approaches combine skills from specialized teachers (Rusu et al., 2015). For social navigation, non-optimal teachers (e.g., PID planners) can be combined with RL, accelerating training by switching to the higher Q-value source (Xie et al., 2018).

#### 3.5.5 Sim-to-real

Sim-to-real transfer for navigation tackles the challenge of adapting a simulation-trained policy to perform reliably in real-world environments. Achieving sim-to-real transfer requires a highly realistic simulator (refer to Section 3.2.2) and the implementation of techniques like domain randomization and domain adaptation. These techniques operate at different levels: scenario-level randomization and adaptation (see Appendix for details) modify various aspects of the simulated environment, while sensor-level noise enables the policy to handle discrepancies in real-world sensor data. *Domain adaptation* adjusts simulation-trained models to real-world domains. For RGB data, this uses real-world samples and methods like discrepancy minimization, adversarial alignment, or reconstruction methods for feature alignment (Wang and Deng, 2018). For depth sensors, techniques such as depth completion and refinement address real-world limitations, improving consistency with simulated data (Khan et al., 2022). *Domain randomization* narrows the sim-to-real gap by introducing simulated variability, allowing policies to generalize to real-world conditions (Tobin et al., 2017). For RGB inputs, this includes varying visual features to simulate lighting and color changes (Anderson et al., 2021); for depth sensors, it involves adding noise, occlusions, warping, and quantization (Muratore et al., 2022; Thalhammer et al., 2019). Active domain randomization further improves robustness by focusing on model-effecting variations (Mehta et al., 2020; Zakharov et al., 2019).

### 3.6 Navigation model evaluation

Evaluating social navigation policies requires a robust approach to ensure reliable and safe robot operation in human environments. This section covers policy evaluation by outlining real-world experiments that validate a robot’s capabilities in realistic, dynamic settings and by presenting metrics that offer structured, quantifiable insights into both navigation performance and social compliance. For a more comprehensive overview of social navigation evaluation, see Francis et al. (2023) and Gao and Huang (2022).

#### 3.6.1 Real-world experiments

Evaluating social navigation policies in real-world settings is crucial for assessing their robustness, adaptability, and social acceptability. Experiments typically fall into three categories: experimental demonstrations, lab studies, and field studies (Mavrogiannis et al., 2023). Experimental demonstrations offer proof-of-concept with limited reproducibility (Chen et al., 2017b; Chen C. et al., 2019), while lab studies provide structured, repeatable tests in controlled environments with systematic reporting (Tsai and Oh, 2020; Mavrogiannis et al., 2019). Field studies are the most comprehensive, deploying robots in public spaces among uninstructed pedestrians (Kato et al., 2015; Kim and Pineau, 2016). Real-world evaluations combine quantitative metrics with qualitative observations, such as participant feedback or questionnaires, to assess social adaptability and compliance (Pirk et al., 2022).

#### 3.6.2 Metrics

Navigation and social navigation metrics provide a structured framework to assess robot performance in crowded environments. Traditional navigation metrics assess robots’ fundamental abilities such as reaching targets and avoiding obstacles, while social navigation metrics focus on interactions with humans, including maintaining personal space and minimizing disruptions to bystanders. Together these metrics, as detailed in Table 9, guide the development of navigation systems that achieve task objectives efficiently while adhering to socially appropriate behaviors, promoting safer and widely accepted robot deployments.

**TABLE 9:** Navigation and social navigation metrics.

Metric	Description
Success	Goal-reaching success rate
Timeout	Exceeded time limit runs
Path Length	Total path distance
Run Time	Completion time
SPL (Anderson et al., 2018b)	Path efficiency-weighted success
Static Obstacle Collision	Static obstacle collisions count
Velocity Metrics	Min, max, and avg. velocity
Speed efficiency (Fraichard and Levesy, 2020)	Ratio of nominal and actual speed
Acceleration Metrics	Min, max, and avg. acceleration
Jerk Metrics	Min, max, and avg. jerk
Obstacle Distance	Min, max, and avg. distance to obstacle
Path Irregularity (Guzzi et al., 2013)	Total deviation from a straight path
Topological complexity (Mavrogiannis et al., 2018)	Amount of entanglement within trajectory
Path Efficiency	Actual vs. straight-line ratio
Failure To Progress (Francis et al., 2023)	Goal progress failure over time period
Human Collision	Number of collisions with humans
Social Distance	Min, max, and avg. distance to humans
Min Time To Collide (Francis et al., 2023)	Min time to projected human collision
Crowd Density	Mean/max crowd density around robot
Virtual Collision (Paez-Granados et al., 2022)	Virtual boundary violations count
Personal Space (Wang et al., 2022c)	Avg time in minimum personal space
Legibility (Dragan et al., 2013)	Goal matches human expectation given robot motion
Predictability (Dragan et al., 2013)	Motion matches human expectation given known goal
Projected Path (Wang et al., 2022c)	Avg duration of path overlap with pedestrian
Following Rate (Puig et al., 2023)	Steps with maintained distance in human-follow task
SPS (Puig et al., 2023)	Path-weighted finding success in human find and follow task

## 4 Social navigation benchmarking

This section benchmarks state-of-the-art social navigation planners from 7 categories, assessing their performance in realistic and challenging scenarios. We achieve efficient and consistent training and evaluation processes by leveraging GPU-based simulation. Additionally, planners are adapted to handle static obstacles such as walls, as most planners only process human positions. We benchmark each planner over 6 scenarios to provide insights into the strengths, limitations, and real-world applicability.

### 4.1 Benchmark setup

A significant challenge in learning-based robotics, including social navigation, is the demanding computational cost of training and evaluation. To address this, we developed a benchmark that leverages GPU parallel computing to accelerate simulation and computation, significantly reducing training time and enabling more extensive experimentation and efficient benchmarking of social navigation planners.

**FIGURE 3:**
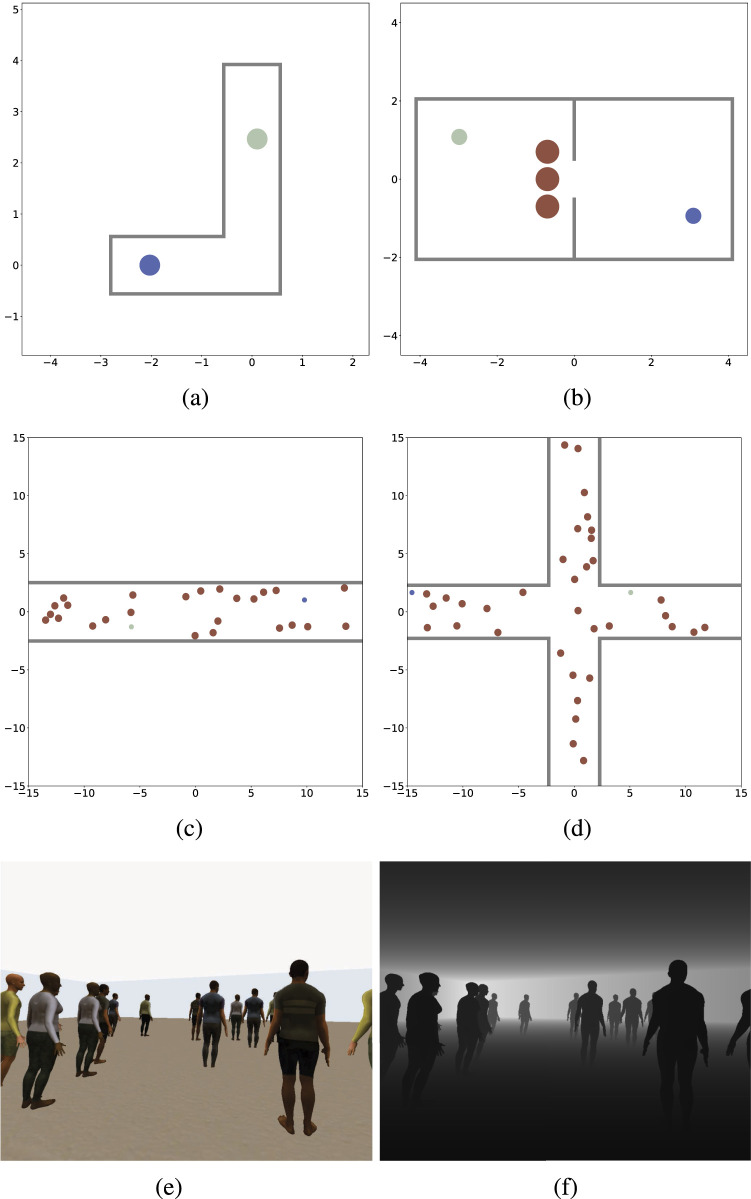
Top-down illustrations of the navigation scenarios **(a–d)**, where the robot is shown in blue, the goal in green, and humans in red. Example RGB and depth images from the benchmark rendered using Habitat Sim are shown in **(e,f)**.

The benchmark comprises three main components: kinematic motion simulation, sensor simulation, and crowd behavior modeling. Kinematic simulation is fully implemented on the GPU, including all computations for rewards and metrics, allowing efficient calculation of agent positions with respect to the map and robot frame. Sensor simulation is also performed on the GPU using Habitat Sim (Savva et al., 2019), which supports RGB and depth camera emulation (see Figures 3e,f), and we generate 2D LiDAR observations via ray casting. The Habitat 3.0 (Puig et al., 2023) codebase further enables photorealistic rendering of 3D moving humans at high frame rates, achieving around 600 FPS for crowds of 40 humans. Existing crowd behavior models are primarily CPU-based, relying on well-established libraries. For diversity and robustness, we incorporate two models: SFM, using the implementation from Gao (2025) with parameters from Helbing et al. (2005), and ORCA, using the implementation from Stüvel (2025) and parameters based on Chen C. et al. (2019).

To enhance training efficiency and success, we employed curriculum learning during training. This technique gradually increases the difficulty of the scenarios as the robot improves. Initially, training focuses on less challenging configurations. As the training progresses, parameters such as crowd density and goal distance are systematically increased. Additionally, training focuses on scenarios where the robot performs poorly, ensuring that the robot performs well across all scenarios.

During evaluation, scenario parameters, including crowd density, goal distances, and map complexity, are randomly and uniformly sampled to ensure diverse testing conditions.

### 4.2 Benchmark scenarios

Benchmark scenarios, illustrated in Figures 3a–d, are designed to comprehensively evaluate social navigation by simulating a range of real-world challenges robots may encounter in crowded indoor and outdoor environments (Gao and Huang, 2022; Francis et al., 2023; Stratton et al., 2024). The robot’s start and goal positions, environment size, and crowd density are randomized within defined bounds, with safety constraints to avoid infeasible or unsafe initializations. six representative scenarios are included: a static scenario with only obstacles to test navigation in narrow spaces; a doorway scenario evaluating interactions at chokepoints (Singamaneni et al., 2022); a corridor scenario capturing integration into unidirectional or bidirectional crowd flows; an intersection scenario representing complex areas where two flows meet; and open space scenarios that simulate unconstrained environments using both random and data-driven human motion, incorporating realistic pedestrian behavior from ETH (Pellegrini et al., 2009) and UCY (Lerner et al., 2007) datasets.

### 4.3 Benchmark planners

To evaluate social navigation strategies, we selected planners based on relevance, novelty, performance, and available implementations. The benchmark features three baselines and six learning-based planners, each covering a distinct social navigation category, along with an imitation-learning method. Since many learning-based planners do not natively handle static obstacles, we extend them with a LiDAR network (Fan et al., 2020), ensuring fair evaluation in environments with both dynamic and static obstacles.

#### 4.3.1 Baseline planners

The baseline planners include ORCA (Van den Berg et al., 2008), the SFM (Helbing and Molnar, 1995), and DWA (Fox et al., 1997). They serve as classical foundations for comparison with advanced methods. Each is given privileged access to the map layout and all human positions, ensuring optimal performance under ideal conditions.

#### 4.3.2 End-to-end planner

The end-to-end planner is based on the RL policy from Fan et al. (2020), which processes recent 2D LiDAR scans with a 1D CNN, combines them with the robot’s state, and uses an MLP for action selection. Due to its suboptimal performance, we adopt an RNN-enhanced architecture (Hoeller et al., 2021), where the CNN output and robot state are fed into a GRU network, improving results. This end-to-end model learns navigation directly from sensor data, without using human state information.

#### 4.3.3 Imitation learning-based planner

We implement Behavioral Cloning (BC) for imitation learning, offering a simple alternative to methods like GAIL (Ho and Ermon, 2016) without needing simulated environments. Trained on 35,000 successful human attention-based planner episodes, matching the planner’s performance would signal robust generalization from real-world data. The network architecture mirrors the human attention-based planner.

##### 4.3.3.1 Human position-based planner

The GA3C-CADRL (Everett et al., 2018) planner uses an actor-critic policy with an LSTM to process human positions and velocities. We extend it with a LiDAR network (Fan et al., 2020) for static obstacle handling, enabling navigation in mixed environments. The LSTM input is zero-padded, and in scenarios without humans, the LSTM layer is skipped.

#### 4.3.4 Human attention-based planner

The SARL planner (Chen C. et al., 2019) employs an attention-based network to model robot-human attentions. We extend the original value network to an actor-critic framework and add a LiDAR network (Fan et al., 2020) for static obstacle handling. Unlike Liu et al. (Liu L. et al., 2020), which switches between separate policies for human and non-human scenarios, our approach uses a learned embedding to pad human input when no humans are present.

#### 4.3.5 Human prediction-based planner

The prediction-based planner adapts the RGL model (Chen C. et al., 2020), integrating robot state and LiDAR input to predict human trajectories in the robot frame. These predicted trajectories are processed by an actor-critic policy, following the SARL planner (Chen C. et al., 2019), to handle fixed-size trajectories. When no humans are present, a learned embedding pads the input for consistency.

#### 4.3.6 Safety-aware planner

Inspired by Linh et al. (2022), the safety-aware planner combines ORCA (Van den Berg et al., 2008) for static environments and the human attention-based planner for dynamic settings, using a policy switcher based on obstacle proximity. This hybrid approach balances safety and efficiency by adapting to both static and human-dense scenarios.

### 4.4 Results

**FIGURE 4:**
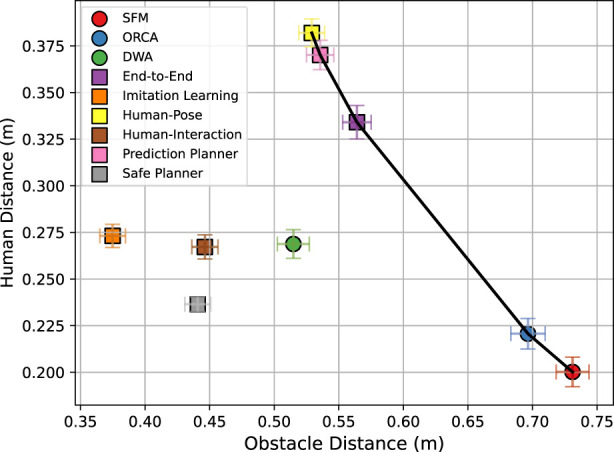
Comparison success rate of of planners based on average minimum obstacle distance and minimum human distance.

Across six scenarios, learning-based planners consistently outperform model-based methods. In terms of success rate and safety, many of these learned policies consistently outperform traditional approaches. Unlike model-based planners, which prioritize obstacle avoidance, learning-based planners tend to emphasize maintaining a safe distance from humans, as illustrated in Figure 4.

In the static scenario, all methods avoid collisions, so success rate and runtime distinguish performance. Model-based planners like ORCA achieve high success but are slower, while learning-based planners are overall faster, sometimes at the expense of a higher timeout rate. Imitation Learning struggles to generalize here. In the doorway scenario, where human-robot interactions are frequent, learning-based planners adapt better, leading to safer navigation and fewer collisions.

In the corridor scenario, both model-based and learning-based planners perform comparably, managing high success rates, efficiency, and safety distances. In contrast, in the intersection scenario, learning-based methods, particularly the prediction-based planner, achieve higher success rates.

**FIGURE 5:**
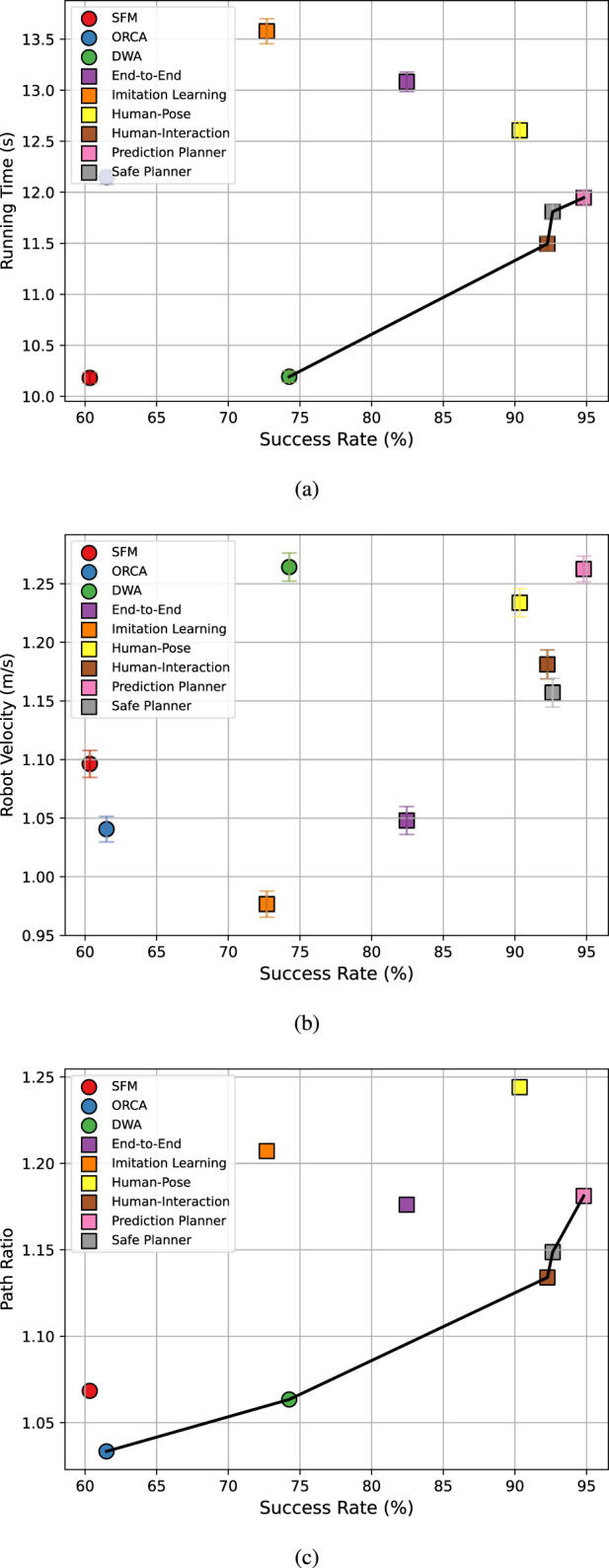
Average of planners based on success rate of each planner versus **(a)** running time, **(b)** robot velocity, and **(c)** path ratio.

In the open space random scenario, learning-based planners achieve higher success rates and smoother navigation by adapting to dynamic human movement, reducing congestion. Model-based methods, while faster, incur more collisions due to riskier behavior. This pattern holds across most scenarios as shown in Figures 5a,c. In the open space data-driven scenario, learning-based planners remain safer while matching the running times of model-based approaches.

Among learning-based methods, end-to-end RL is notably conservative and prioritizes safety. Imitation Learning generalizes well in open spaces but struggles in constrained settings. The human position-based planner excels in open areas through direct spatial awareness, while the Human attention-based planner adapts best in crowded environments using attention mechanisms. The safety-aware planner balances efficiency and safety but remains limited by its learning-based component. The prediction-based planner, with its prediction module and expressive architecture, achieves the highest overall success rate and velocity, as shown in Figure 5b.

## 5 Discussion and future directions

Despite the progress in social navigation, several challenges remain for learning-based social navigation to achieve safe and reliable real-world deployment. We organize this discussion around three priority levels: foundational requirements for safety and robustness, socially aligned behaviors for human acceptance, and capabilities that improve transparency and versatility.

### 5.1 Foundations for safety and realism

Ensuring safe and robust navigation is the highest priority for real-world deployment.

#### 5.1.1 Safety and robustness

Most safety-oriented planners, like multi-policy approaches (Sun et al., 2019; Katyal et al., 2020; Fan et al., 2020), assume that reducing speed enhances safety, but this is not always valid; rapid maneuvers may be needed in dynamic, crowded settings. Relying solely on speed reduction can compromise safety in complex environments. Similarly, several human-prediction-based methods (Yao et al., 2024; Zhu et al., 2025) primarily forecast human motion without explicitly modeling the robot’s influence on the crowd, which limits their ability to generate safe and adaptive plans. Instead, planners should learn context-aware safe behaviors, adjusting speed as needed and responding to emergencies, to achieve both safety and efficiency without unnecessary conservativeness.

#### 5.1.2 Scenario diversity and generalization

A major limitation to model generalizability is the limited diversity of training scenarios. Future benchmarks should incorporate a wider range of realistic, data-driven scenarios reflecting true pedestrian distributions and start-goal configurations. Long-term crowd tracking datasets similar to, or even larger than ATC (Brščić et al., 2013), which capture varied environments in a shopping mall, can help provide such diversity.

#### 5.1.3 Physics and sensor realism

Physics simulators range from simple kinematic to detailed dynamic models, with high-fidelity simulation improving sim-to-real transfer and enabling robot-specific planners that can integrate low-level control, such as direct wheel velocities. Likewise, accurate sensor simulation enhances robustness; while most simulators use generic models for simplicity (Inc D, 2025), sensor-specific models that replicate real-world parameters and noise can significantly improve generalization and sim-to-real performance.

#### 5.1.4 Realistic crowd simulation

Most crowd simulation methods focus on human-human and human-obstacle interactions, but accurately modeling human-robot interactions remains a challenge. Some approaches ignore the robot’s presence (Chen Y. et al., 2020; Dugas et al., 2021), leading to unrealistic and overly conservative behavior, while others treat robots as humans or add randomness for robustness (Chen C. et al., 2019; Stratton et al., 2024). However, these do not fully capture the diverse ways humans respond to robots, which depend on robot-specific factors like size, shape, and movement. More advanced crowd models that reflect these characteristics are needed for realistic social navigation simulation.

#### 5.1.5 Robust evaluation

Advancing social navigation requires robust benchmarking methods that can accurately represent the planner’s performance. Key directions include adopting realistic crowd simulation, conducting real-world evaluations, and refining social metrics (Francis et al., 2023; Gao and Huang, 2022). Automated, objective real-world evaluation frameworks are increasingly important, as subjective user feedback is impractical to standardize. Future evaluations could use objective, non-verbal indicators, such as body language or facial expressions to better assess human comfort and social acceptance, ensuring planners are both effective and socially appropriate.

### 5.2 Social alignment and preferences

Beyond safety, social navigation must align with human expectations and adapt to cultural and individual differences.

#### 5.2.1 Social norms and compliance

Social norms are informal rules guiding behavior in shared spaces, extending beyond collision avoidance and proxemics (Hall, 1963). For instance, smoothly avoiding social groups is addressed by some crowd prediction methods (Bisagno et al., 2018; Fernando et al., 2019), but is incorporated into only a few navigation algorithms (Bhaskara et al., 2023). Other norms, such as culturally specific conventions (Chen et al., 2017c), are context-sensitive and not universal, suggesting the value of learning social norms directly from large-scale crowd data rather than relying solely on handcrafted heuristics. Vision-language models (VLMs) open an additional pathway by enabling robots to ground these norms in natural language, reason about complex social contexts, and even communicate intentions to humans in interpretable ways. Effective social navigation will likely require a combination of data-driven norm learning and VLM-based reasoning, alongside intention communication that may be verbal (Dugas et al., 2020; Nishimura and Yonetani, 2020) or conveyed through non-verbal cues, as highlighted in autonomous vehicle research (Habibovic et al., 2018).

#### 5.2.2 Human preferences

Social navigation is not a one-size-fits-all solution. Individuals and crowds vary in preferred comfort distance, speed, and interaction style. Future work should emphasize preference-aware navigation, where robots learn and adapt to individual users or cultural groups, potentially combining reinforcement learning with preference learning, feedback, or large language models that capture human expectations and feedback. Although current approaches consider human preferences during training (Choi et al., 2020), accommodating post-deployment feedback and achieving continuous learning remain open challenges.

### 5.3 Transparency and reasoning

To ensure long-term acceptance, learning-based systems must be interpretable, communicative, and capable of reasoning based on context.

#### 5.3.1 Explainability and transparency

A major challenge in learning-based planners is the difficulty of interpreting the reasoning behind their decisions, which is often referred to as *explainability* (Vouros, 2022). Integrating explainability improves user trust, allows better debugging, and clarifies the decision-making process. Several techniques exist, such as *saliency maps*, which visually indicate influential regions within image-based inputs (Huber et al., 2021), and approaches that provide verbal explanations for their decisions (Dugas et al., 2020). Integrating these explainability methods into learning-based social navigation can create more transparent, interpretable, and user-friendly systems.

#### 5.3.2 Social vision-language navigation

Recent advances in vision-language navigation (VLNs) (An et al., 2022) highlight opportunities to enrich social navigation with multimodal reasoning capabilities and improve functional versatility. Beyond instruction following (Anderson et al., 2018a), VLNs can support a wide range of tasks such as visual question answering (Wu et al., 2024), describing social situations, or embodied dialog (Hahn et al., 2020). Social VLN could allow robots to interpret human intent, infer social norms from linguistic context, and communicate their own decisions in interpretable ways.
