# Supplementary material for: Social robot navigation: a review and benchmarking of learning-based methods
Source: Front Robot AI. 2025 Dec 11;12:1658643. doi: 10.3389/frobt.2025.1658643 (PMC12739477; doi:10.3389/frobt.2025.1658643)
Supplement: Supplementary file 1 [file Supplementaryfile1.pdf]

APPENDIX

The appendix provides technical background, training environment details, and experimental results for social navigation. The background section covers key concepts of social navigation, including local and global planners, as well as learning-based approaches. The training environment section discusses collision avoidance training, crowd behavior modeling, map design, and crowd goal assignment. The experiment section presents benchmark experiments across various categories of social navigation planners, including further implementation details.

1 BACKGROUND

This section outlines the technical foundations essential to learning-based social navigation. It emphasizes localization and mapping techniques to ensure accurate positioning, as well as global and local planning for map-level path planning and real-time path execution, respectively. We outline the fundamentals of reinforcement learning (RL) and imitation learning, as they serve as the foundation for learning-based planning. Additionally, neural network architectures such as Recurrent Neural Networks (RNNs), Graph Neural Networks (GNNs), and Transformers play essential roles in prediction and decision-making within social navigation.

1.1 Navigation

Robot navigation refers to the ability of mobile robots to move autonomously from one location to another within their environment. Navigation relies on *localization*, which enables the robot to determine its precise position, and *mapping*, which builds a detailed representation of the surrounding area.

Navigation or *motion planning* builds upon localization and mapping by directing the robot from a starting point to a destination while considering kinematic constraints and obstacle avoidance. In a 2D planning space, motion planning is divided into path planning and low-level control. *Path planning* involves determining a sequence of waypoints or an initial route to reach the goal, encompassing both global planning and local planning. Global planning utilizes a known map to establish a broad and efficient path that avoids static obstacles, whereas local planning operates in real-time to execute the global path and make swift adjustments in response to dynamic obstacles or unexpected changes. Local planners capable of handling human crowds fall under the category of social navigation. *Low-level control* translates the planned actions into precise motor commands, ensuring the robot accurately follows the intended path.

1.1.1 Localization and Mapping

Simultaneous localization and mapping (SLAM) enables a robot to create a map of its environment while simultaneously determining its position within that map. SLAM techniques vary based on the underlying algorithm, sensor type, and map representations, such as landmark-based maps that utilize distinct features or volumetric maps that capture detailed 2D or 3D layouts (1).

Filter-based SLAM, like FastSLAM (2), employ Kalman and Particle filters to update maps using sensory input, making them well-suited for 2D LiDAR mapping. *Graph-based* SLAM, such as RGB-D based ORB-SLAM3 (3), construct pose graphs that optimize map accuracy through graph optimization techniques. *Deep learning-based* SLAM, including DeepFactors (4), leverage neural networks to estimate depth from RGB images, enhancing mapping precision in complex environments. Furthermore, *active SLAM* (ASLAM) automates map construction by extending SLAM with planning strategies to maximize information gain (5).

1.1.2 Global Planning

Global planning is the process of computing an optimal path from a robot's current position to a desired goal by considering the entire mapped environment.

Graph-based Global Planning. One of the earliest approaches in global planning is graph-based methods. These methods involve constructing a graph by generating nodes in a predefined pattern, such as a rectangular grid, and adding collision-free edges to connect them. Algorithms like Dijkstra's algorithm (6) are then used to find the shortest path between nodes systematically, assuming non-negative edge costs. Another popular algorithm is A* (7), which enhances search efficiency by employing heuristics to guide exploration toward the goal. A* has expanded into several variants, including hybrid A* (8), which produces kinematically feasible trajectories, and D* (9), which can dynamically update paths as the environment changes, thereby avoiding the need for full replanning.

Roadmap-based Global Planning. *Roadmap* based planners construct a graph by strategically sampling nodes within free space or along its boundaries to enable efficient navigation. Examples include visibility graphs (10), which connect the vertices of polygonal obstacles based on line-of-sight, thereby providing the shortest paths, though often close to obstacles. Voronoi diagrams (11) partition the space into regions based on proximity to a set of points, creating paths that maximize clearance from obstacles for safer navigation. Additionally, dynamic roadmaps (12) adapt the graph structure in response to changes in a dynamic environment, adjusting to moving or newly introduced obstacles.

Sampling-based Global Planning. Sampling-based methods utilize randomized tree structures or graph-based techniques instead of structured approaches, enhancing scalability and efficiency in complex environments. A well-known example is the Probabilistic Roadmap (PRM) (13), which creates a network of randomly sampled, collision-free nodes connected by edges. Algorithms like Dijkstra or A* can then search this network for the shortest path. A key advantage of PRM is that once created, the roadmap can be reused for multiple goals in the same environment, making it efficient for static settings. Another approach is the rapidly exploring random tree (RRT) (14), which builds a tree from the start point towards the goal by extending edges from the nearest existing node to randomly sampled points. Variants like RRT* (15) improve path quality with heuristics, while RRTX (16) dynamically updates the tree to handle changes in the environment. Real-time RRT* (17) incorporates time-step adjustments, making it suitable for time-sensitive applications. Although effective for local planning, RRTX and Real-time RRT* react more slowly than traditional local planners due to their reliance on random sampling.

Learning-based Global Planning. Learning-based global planning approaches aim to enhance traditional planners by integrating machine learning techniques to improve efficiency and adaptability in complex environments. OracleNet (18) is an RNN-based planner that excels in high-dimensional configuration spaces but requires retraining for each new environment, making it suitable for static yet complex settings. Other methods focus on optimizing RRT*. For example, Palmieri and Arras (19) introduce a time-to-reach (TTR) function that estimates the kinodynamic travel time between states, serving as an efficient distance metric in RRT* to improve accuracy. Chiang et al. (20) extend this concept with a reachability estimator that predicts the time required for an RL-based local planner to travel between points. Wang et al. (21) propose a CNN-based model that generates a distribution map of the environment, guiding RRT* sampling based on learned spatial probabilities. For a more flexible architecture, MPNet (22) combines an encoder network that captures environment features with a planning network that predicts iterative path steps, utilizing active continual learning to request expert guidance only when uncertainty is high. Similarly, Neural A* (23) is a neural-guided A* approach where an encoder generates a guidance map used by a differentiable A* search to obtain optimal paths.

Path Segmentation. The global planner's path is transmitted to the local planner either as a

continuous path (24) or as discrete waypoints. Waypoints enhance flexibility by enabling the robot to proceed to the next waypoint once it is within a specified distance, although this may compromise motion smoothness (25). Methods such as PRM generate a sparse set of nodes that naturally serve as waypoints (26), while other approaches establish waypoints at fixed intervals (25). Advanced techniques add waypoints based on direction changes, resulting in denser spacing in complex areas and sparser in simpler regions (27).

Costmaps. Navigation costmaps typically represent static elements such as walls and furniture within an environment. However, in social navigation, costmaps are often enhanced to encode dynamic, human-centered information that aids both local and global planning. For example, configuration space (c-space) costmaps extend beyond traditional obstacles by assigning cost values based on feasible robot positions and orientations. Layered costmaps, introduced by Lu et al. (28), allow separate cost layers for different obstacles or constraints to be combined into a master costmap, supporting flexible adaptation to human motion. Guo et al. (29) utilize Gaussian processes to model humans on a costmap, while Kollmitz et al. (30) predict human trajectories by creating multiple costmaps for future time steps that inform an A*-based local planner. Fang et al. (31) enhance this approach by incorporating group dynamics in global planning, and FlowBot (32) treats crowds as a pseudo-fluid, generating flow maps with Dijkstra's algorithm to produce minimum-time paths through social spaces. Costmaps are then integrated with global planners for improved navigation. For instance, Jaillet et al. (33) introduce a transition-based RRT (T-RRT) on c-space costmaps, which has been adapted for mobile manipulators to avoid humans by creating dynamic 3D costmaps around them (34).

1.1.3 Local Planning

Local planning is the process of generating immediate, short-term motion commands to navigate around nearby obstacles and dynamically adapt to changes in the immediate environment to reach a nearby goal. Local planning approaches are generally categorized into two main types (35): reactive-based planning and learning-based planning. Reactive-based methods can be further subdivided into three groups: field-based, velocity-based, and optimization-based approaches.

Field-based Planning. Potential field methods, such as the artificial potential field (APF) (36), model the goal as an attractive force and obstacles as repulsive forces, thereby creating a field that guides the robot toward the goal while avoiding obstacles. To better handle dynamic obstacles, evolutionary APF (37) provides adaptability to changing environments. However, APF has limitations, particularly in dealing with local minima and navigating narrow passages. To overcome these challenges, variations like the virtual force field (VFF) (38) and vector field histogram (VFH) (39) were introduced. VFH enhances navigation by constructing a local occupancy grid based on recent sensor data, enabling dynamic obstacle avoidance. VFH+ (40) and VFH* (41) further improve upon this by incorporating the robot's motion constraints and search heuristics, respectively, facilitating smoother navigation in complex environments.

Velocity-based Planning. Velocity-based methods generate safe velocity commands by analyzing the space around the robot to identify collision-free velocities. Velocity obstacles (VOs) (42) are a fundamental approach that identifies velocities likely to cause collisions within a set time horizon, enabling the robot to select speeds outside of these VOs to avoid collisions. For multi-robot scenarios, VO has been extended to reciprocal velocity obstacles (RVO) (43), which assumes that each robot will choose a velocity that avoids collisions by considering the movements of others. Optimal reciprocal collision avoidance (ORCA) (44) further enhances RVO by balancing collision avoidance responsibilities among robots and optimizing the safe velocity space.

Optimization-based Planning. Optimization-based planners generate optimal control commands

by minimizing a cost function at each step, ensuring that the path satisfies specific criteria such as safety, efficiency, and obstacle avoidance. A prominent example is the model predictive control (MPC), which continuously computes optimal commands by predicting the robot's dynamics and solving an optimization problem at each time step. MPC minimizes a cost function that accounts for obstacles, kinematic constraints, and reaching the goal. It operates on a receding horizon, executing only the first action of the optimized sequence before recalculating with updated information. This approach makes MPC highly adaptive to dynamic environments, although it is computationally demanding. One of the earliest methods that can be considered an adaptation of MPC with discrete optimization is the dynamic window approach (DWA) (24). DWA computes the minimum cost over a predefined set of possible actions by evaluating the robot's dynamic constraints and selecting the optimal velocity commands for differential drive robots. Variants such as the global dynamic window (GDW) approach (45) extend this concept to facilitate planning in unknown and dynamic environments, while the time varying dynamic window (TVDW) (46) computes future obstacle trajectories to enhance navigation. Another optimization-based technique is the elastic bands method (47), which deforms a collision-free path by adding *bubbles* around it that adjust in response to obstacles. Internal forces contract the path for efficiency, while external forces push it away from obstacles. An extension of this method, timed elastic bands (TEB) (48), incorporates time into the optimization process and considers the robot's kinodynamic constraints, making it well-suited for dynamic and real-world environments.

Learning-based Planning. Learning-based methods for local planning can be categorized into evolutionary algorithms, fuzzy control, and artificial intelligence (35). Evolutionary algorithms are inspired by natural selection, utilizing mechanisms such as mutation, crossover, and survival of the fittest to iteratively enhance a population of candidate solutions toward an optimal outcome. These algorithms are robust and capable of addressing complex local planning challenges, such as obstacle avoidance and path optimization (49, 50, 51). However, they are computationally intensive and may face difficulties with convergence. Fuzzy control employs fuzzy logic to manage uncertainty and approximate information, which is advantageous in scenarios where precise data is either unavailable or unreliable. For instance, fuzzy control can assist robots in environments with incomplete obstacle information by making decisions based on degrees of truth rather than binary logic (52, 53). While effective at handling ambiguity, fuzzy control can be computationally demanding, may encounter convergence issues, and tends to scale poorly in more complex environments. Artificial intelligence methods, including RL, address these challenges by leveraging advanced learning techniques, detailed in Section 1.2.

1.2 Learning-based Planning

Sequential planning problems are often modeled as Markov decision processes (MDPs), which provide a formal framework for decision-making in uncertain environments. Solving MDPs typically involves two approaches: planning-based and learning-based methods. Planning-based methods, such as value iteration and policy iteration, utilize dynamic programming techniques to compute an optimal policy by iteratively updating values or policies based on a known model of the environment's dynamics. However, these methods require prior knowledge of the environment and struggle with continuous state spaces.

In contrast, learning-based methods, particularly reinforcement learning (RL), address MDPs without assuming complete knowledge of the environment. RL algorithms learn an optimal policy, denoted as π, through trial-and-error interactions, continuously adapting based on feedback from the environment. Deep RL (DRL) extends RL by employing neural networks to approximate the policy function, represented as $\pi(\cdot \mid \theta)$, where θ denotes the neural network parameters. Given DRL's widespread adoption, the term RL is used interchangeably with

DRL. Additionally, imitation learning substitutes RL by deriving policies from expert demonstrations using supervised learning, thereby eliminating the need for extensive trial-and-error.

1.2.1 Markov Decision Process

A Markov decision process (MDP) is defined as a tuple $\mathcal{M} = (\mathcal{S}, \mathcal{A}, T, d_0, r, \gamma)$, where $s \in \mathcal{S}$ is the set of states, and $a \in \mathcal{A}$ is the set of actions. Both states and actions can be discrete or continuous. The transition function $T(s_{t+1} \mid s_t, a_t)$ is a conditional probability distribution that describes the system dynamics. $d_0(s_0)$ is the initial state distribution, r is the reward function that maps state-action pairs to scalar rewards $\mathcal{S} \times \mathcal{A} \to \mathbb{R}$, and $\gamma \in [0, 1]$ is the discount factor, determining the weighting of future rewards.

POMDP. A partially observable Markov decision process (POMDP) is defined as a tuple $\mathcal{M} = (\mathcal{S}, \mathcal{A}, \mathcal{O}, T, d_0, E, r, \gamma)$, where $(\mathcal{S}, \mathcal{A}, T, d_0, r, \gamma)$ are identical to those in an MDP. Here, $\mathcal{O}$ represents the set of observations $o \in \mathcal{O}$ that the agent receives instead of the full state s. The emission (or observation) function $E(o_t \mid s_t)$ defines the probability distribution over observations given the current state. Most real-world planning problems are modeled as POMDPs because agents often lack complete information about the environment's full state. Instead of explicitly learning the emission function E, most RL approaches handle partial observability by directly learning an optimal policy $\pi^*(o_{0:t})$ based on sequences of observations. This allows the problem to be treated similarly to an MDP, focusing on learning the optimal policy from observations rather than complete state information. In practice, the sequence of observations is typically managed using an RNN or a neural network architecture that processes the last set of observations as input (54).

Policy. The objective is to learn a policy, which defines a distribution over actions given observations, denoted as $\pi(a_t \mid o_t)$, or the observation history $\pi(a_t \mid o_{0:t})$. Policies can be deterministic or stochastic. Deterministic policies output a specific action for each state or observation, while stochastic policies assign probabilities to each possible action. For discrete states and actions, policies are often represented in tabular form. For continuous states or actions, it is more common to use parametric models such as linear models or neural networks to approximate the policy function.

Objective Function. The objective function maximizes the expected discounted cumulative reward over a trajectory τ,

$$J(\pi) = \mathbb{E}_{\tau \sim p_\pi(\tau)} \left[\sum_{t=0}^{h-1} \gamma^t r(s_t, a_t) \right] \quad (1)$$

where $\tau = (s_0, a_0, \ldots, s_{h-1}, a_{h-1})$ represents a trajectory, and h is the planning horizon or the time step at which the episode terminates. Here, $\gamma \in [0, 1]$ is the discount factor that balances the importance of immediate versus future rewards, and $p_\pi(\tau)$ denotes the distribution of trajectories induced by the policy π.

Value Function. Value functions estimate the expected future rewards for states or state-action pairs, which is essential in RL value-based methods and guides policy improvement in policy-based methods. They represent the expected cumulative reward when following a given policy π from a given state. The state value function $V^\pi(s_t)$ estimates the expected reward starting from state s_t and following policy π:

$$V^\pi(s_t) = \mathbb{E}_{\tau \sim p_\pi(\tau \mid s_t)} \left[\sum_{t'=t}^{h} \gamma^{t'-t} r(s_{t'}, a_{t'}) \right] \quad (2)$$

The action value function $Q^\pi(s_t, a_t)$ estimates the expected reward starting from state-action pair (s_t, a_t) and following policy π:

$$Q^\pi(s_t, a_t) = \mathbb{E}_{\tau \sim p_\pi(\tau \mid s_t, a_t)} \left[\sum_{t'=t}^{h} \gamma^{t'-t} r(s_{t'}, a_{t'}) \right] \quad (3)$$

1.2.2 Reinforcement Learning Algorithms

RL algorithms focus on learning how to map states to actions to maximize a numerical reward signal (55). RL algorithms address MDPs by learning a policy π directly through interaction with the environment. These algorithms are typically divided into three categories: value-based methods, which estimate the optimal value function; policy-based methods, which directly learn the optimal policy; and model-based methods, which learn or utilize a model of the environment to simulate future states and rewards. Additionally, RL algorithms also differ in data requirements: on-policy algorithms use data from the latest policy for consistency, while off-policy algorithms allow data from any policy, offering more flexibility.

Value-based RL. Value-based algorithms such as deep Q-learning (DQN) (56) and its variants, double DQN (57), dueling DQN (58), and distributional DQN (59), learn the action-value function $Q(s, a)$ and derive a deterministic policy using the greedy approach $\pi(s) = \arg\max_a Q(s, a)$. The Q-learning update rule is $Q(s_t, a_t) = r_t + \gamma \max_{a'} Q(s_{t+1}, a')$. DQN is off-policy because it updates the policy without requiring that a_t be taken under the current policy π. However, DQN and other value-based methods inherently struggle with continuous action spaces, as the maximization over a continuous set of actions becomes intractable. This limitation restricts their applicability in tasks like navigation, where actions often involve continuous velocities.

Policy-based RL. Policy-based methods directly optimize the policy $\pi(a \mid s; \theta)$, where θ represents the policy parameters, by performing gradient ascent on the expected cumulative reward $J(\pi)$. One of the earliest policy gradient methods, REINFORCE (60), updates θ by computing $\nabla_\theta \log \pi(a_t \mid s_t; \theta) r_t$, thereby maximizing the likelihood of actions that yield high rewards. Policy-based methods can effectively handle continuous action spaces and operate as either on-policy or off-policy. However, they are generally less sample-efficient than value-based approaches.

Policy-based RL (On-policy). Actor-critic methods combine policy-based strategies with value functions to improve stability and sample efficiency. Here, the actor represents the policy, while the critic provides an estimated reward signal, typically via the advantage function $A(s, a) = Q(s, a) - V(s)$, which reduces the variance by comparing the state-action value to the state value. Popular on-policy actor-critic algorithms include advantage actor-critic (A2C) and asynchronous A2C (A3C) (61), both of which support parallel training with synchronous and asynchronous updates, respectively. Another class of on-policy methods focuses on maximizing a *surrogate objective* rather than directly maximizing the expected reward. This strategy provides a conservative update mechanism, thereby stabilizing policy changes. Trust Region Policy Optimization (TRPO) (62) achieves this by constraining the Kullback–Leibler (KL) divergence (63) between the new and old policies, ensuring that updates do not deviate excessively from the current policy. Proximal Policy Optimization (PPO) (64) simplifies TRPO by introducing a clipped objective, which makes the algorithm straightforward to implement and more computationally efficient while still maintaining stable policy updates.

Policy-based RL (Off-policy). Off-policy actor-critic methods combine policy optimization with Q-learning to handle continuous action spaces by approximating the action that maximizes the Q-value. Deep deterministic policy gradient (DDPG) (65) is a notable example that extends DQN to continuous action spaces. DDPG employs a deterministic policy to directly maximize the Q-value, enabling it to handle environments where actions are not discrete. Twin delayed deep deterministic policy gradient (TD3) (66) builds upon DDPG by enhancing stability and performance. TD3 introduces double Q-learning with two separate Q-networks to reduce overestimation bias and implements delayed policy updates, which leads to more accurate Q-value

estimations and improved learning stability. Soft actor-critic (SAC) (67) is another prominent variant that extends DDPG by incorporating a maximum entropy framework. SAC trains a stochastic policy using the reparameterization trick (68), which promotes exploration by encouraging policies to maximize both expected rewards and entropy.

Model-based RL. Model-based RL methods aim to leverage or learn a model of the environment's dynamics to guide decision-making. This dynamics model can be either explicitly provided or learned from data and may operate directly within the state space or a simplified latent space. Once established, the dynamics model can be utilized in two primary ways. It can support model-free RL algorithms by simulating environment interactions (69). Alternatively, techniques such as Monte Carlo tree search (MCTS) (70) and model predictive control (MPC) (71) use the dynamics model to plan and select optimal actions iteratively. This process enables the planner to make continuous adjustments to decisions, hence increasing robustness against potential model inaccuracies. MCTS constructs a state-action tree by iteratively expanding nodes based on a *tree policy* and estimating the value of leaf nodes through simulations using a *rollout policy* (72). It typically employs upper confidence bounds for trees (UCT) (73) to balance exploration and exploitation effectively. As an anytime algorithm, MCTS can provide a viable solution at any stage of the tree expansion, making it well-suited for real-time applications. In contrast, MPC is an optimization-based planner that determines control actions by minimizing a cost function over a predicted horizon using the dynamics model. The optimization problem in MPC can be linear or non-linear, depending on the requirements of the specific application. Both MCTS and MPC utilize a *receding horizon* approach (74), where they iteratively plan over multiple steps but execute only the first action in the planned sequence. Latent state representations are often employed to simplify dynamics modeling when working with complex state spaces, such as those derived from sensor readings (75). Methods like *Predictron* (76), VPN (77), and *MuZero* (78) focus on learning dynamics and value functions directly within a latent space, employing iterative planning to estimate the best action. *World models* (79), *Dreamer* (80), and *SimPLe* (81) leverage latent representations to learn both a dynamics model and a planning policy. Additionally, *MBPO* (82) and *recurrent World models* (83) emphasize accurate short-horizon rollouts; *MBPO* uses ensemble models for robustness, while *recurrent World models* utilize RNNs to capture temporal dependencies across sequences. In model-based social navigation, the state space typically consists of both the robot and humans. The robot's dynamics model is usually predefined, while the crowd's dynamics are inferred using human trajectory prediction models, effectively serving as a dynamics model.

1.2.3 Imitation Learning

Imitation learning enables agents to learn tasks by mimicking expert demonstrations, making it especially useful in scenarios where the reward function is unknown or difficult to specify (84). The primary goal is to replicate the behavior of the expert, and imitation learning methods are broadly categorized into behavior cloning, inverse reinforcement learning, and hybrid approaches that combine elements of both.

Behavior cloning (85) is a supervised learning method that trains a policy by mapping state-action pairs from expert trajectories. While effective with large datasets, it faces challenges with smaller datasets due to compounding errors, a problem known as *covariate shift* (86). This occurs when the model encounters unfamiliar, out-of-distribution states during execution, leading to progressively worsening predictions.

Inverse reinforcement learning (IRL) (87) aims to infer a reward function that explains why the expert's behavior is optimal. The learned reward function evaluates entire trajectories (or episodes), enabling the agent to distinguish between more and less favorable outcomes. However, IRL algorithms are computationally demanding, as they typically require running an RL algorithm to derive a policy

from the inferred reward function. This added complexity can be a drawback, particularly when the primary goal is to directly learn a policy.

Generative adversarial imitation learning (GAIL) (88) is a hybrid method that directly learns a policy from expert demonstrations. Inspired by generative adversarial networks (GANs), GAIL employs two networks: a generator (the policy) and a discriminator. The generator interacts with the environment to produce state-action pairs, while the discriminator's role is to distinguish these from expert demonstrations, providing a reward signal to guide policy updates through model-free RL. GAIL alternates between updating the discriminator and the policy, progressively encouraging the policy to replicate expert behavior. Although GAIL is data-efficient, it is computationally intensive and prone to training instability, similar to challenges faced in GAN-based methods.

Imitation learning has been applied in fields such as autonomous vehicles (89) and robot navigation, by mimicking expert human control. While human driving data are often sufficient for training autonomous vehicles, robot navigation generally depends on model-based planners due to the limited availability of human demonstrations. However, the quality of the learned behavior is fundamentally restricted by the capabilities of the expert model, and adapting the learned policies to different robots can be challenging due to variations in robot dynamics.

1.2.4 Neural Network Architectures

Learning-based planners often leverage neural networks to address the complexities of decision-making in dynamic environments. These networks excel at processing high-dimensional sensory inputs, identifying complex patterns, and modeling the non-linear dynamics inherent in human-robot interactions. The choice of neural network architecture depends on the characteristics of the input data and the specific patterns or relationships the model aims to capture. This section explores commonly used neural network architectures in social navigation, highlighting their distinct advantages and ideal applications.

Recurrent Neural Networks. Recurrent neural networks (RNNs) are a specialized neural network architecture designed to process sequential data by utilizing feedback loops, allowing them to capture temporal dependencies (90). This recurrent structure enables RNNs to retain information across time steps, making them ideal for tasks such as sequence prediction and temporal encoding. A standard, or *vanilla* RNN operates with an input vector x_t and a hidden state vector h_t. At any time step t, the hidden state is computed as $h_t = \sigma(W_{xh}x_t + W_{hh}h_{t-1} + b_h)$, where W and b are the hidden layer's weight and bias parameters, respectively. RNNs are trained using the BPTT algorithm (91). However, vanilla RNNs often struggle with vanishing or exploding gradients, which hinders their ability to capture long-term dependencies in sequences (92). Long short-term memory (LSTM) networks overcome gradient issues in RNNs, enabling the capture of long-term dependencies (93). LSTMs use a *memory cell* to store long-term information and three gates to manage data flow: the *forget gate* discards irrelevant information, the *input gate* integrates new information, and the *output gate* releases information as the hidden state. This design allows LSTMs to handle both short- and long-term dependencies effectively. Gated recurrent units (GRUs) (94) offer a simpler alternative by combining the memory cell and hidden state. It uses a *reset gate* to control the influence of past information and an *update gate* to combine the candidate and current hidden states. GRUs are computationally efficient, perform comparably to LSTMs, and are often preferred in real-time applications. In social navigation, RNNs play two key roles. First, they function as sequence predictors, processing input sequences to forecast the next point, such as predicting human trajectories. Second, they act as sequence encoders, converting variable-length inputs into fixed-size representations. This is particularly valuable for embedding human states for social

navigation, where the number of humans is constantly changing.

Graph Neural Networks. Graph Neural Networks (GNNs) are a class of neural networks designed to process and learn from graph-structured data $G = (V, E)$ by iteratively aggregating and transforming information from neighboring nodes. The primary objective of a GNN is to generate meaningful embeddings for nodes, edges, or the entire graph, which can be applied to tasks such as node classification, link prediction, and graph classification. GNNs operate through *message passing*, where neighboring nodes exchange and aggregate information. Each layer of the network performs one step of message passing, progressively integrating information from more distant nodes. The number of layers determines the depth of a node's computation graph and its ability to capture long-range dependencies. A typical GNN comprises several key components: the message, which represents information exchanged between nodes; the aggregation function, which combines messages from neighboring nodes; the layer connectivity, which defines how layers are connected (e.g., sequential or with skip connections); graph manipulation, which constructs the computation graph; and the learning objective, which guides the training process. Formally, the embedding of a node v at layer l is updated as:

$$h_v^l = \sigma\left(W^l \cdot \text{AGG}\left(\left\{h_u^{l-1} : u \in N(v)\right\}\right) + B^l h_v^{l-1}\right) \tag{4}$$

where W^l, B^l are the weights and bias for layer l, $N(v)$ is the set of neighbors for node v, h_u^{l-1} is the embeddings of neighboring node u from the previous layer, and AGG is the aggregation function. Different GNN algorithms implement this general form with distinct choices in aggregation and message functions. For instance, graph convolutional networks (GCNs) apply a simple mean (degree-normalized sum) aggregation (95), while GraphSAGE extends this by using pooling functions such as max or LSTM-based aggregation (96). Graph attention networks (GATs) enhance flexibility by learning attention weights that prioritize influential neighbors, defining h_v^l with weighted contributions from neighbors (97). In social navigation, GNNs model a crowd as a graph, where humans are represented as nodes, and relationships or proximity define the edges. The objective is to generate node embeddings that capture the interactions and influence of nearby individuals, enhancing planning by incorporating social dynamics.

Transformers. The Transformer model, introduced by Vaswani et al. (98), is a foundational deep learning architecture widely applied in natural language processing and computer vision (99). This sequence-to-sequence model consists of an encoder and decoder, each composed of L identical layers. Encoder layers include a multi-head self-attention mechanism and a feed-forward network, with residual connections and layer normalization to ensure stable training. The decoder incorporates an additional multi-head attention layer to integrate encoder outputs. Positional encoding allows the model to capture the order of sequences. The core of the Transformer model is the attention mechanism, which maps a query Q and a set of key-value pairs K, V to an output. The attention is computed using the scaled dot-product attention:

$$Attn(Q, K, V) = \text{softmax}\left(\frac{QK^T}{\sqrt{D_k}}\right)V. \tag{5}$$

where dividing by key vector dimension $\sqrt{D_k}$ addresses gradient issues in the softmax function. The multi-head attention (MHA) is defined as $MHA(Q, K, V) = \text{Concat}(h_1, ..., h_H)W^O$, where $h_i = Attn(QW_i^Q, KW_i^K, VW_i^V)$, enhances this mechanism by allowing the model to attend to multiple aspects of the input. The outputs from each head h_i are concatenated and projected back to the original dimension. MHA is primarily used as a self-attention mechanism, which computes the relevance of each item in a sequence to all other items (100). This is achieved by setting the input X as the query, key, and value parameters in the attention function,

i.e., $MHA(X, X, X)$. Transformers have been applied to tasks such as human motion prediction (101) and social navigation (102), serving as a more expressive alternative to RNNs. The self-attention mechanism is particularly effective for modeling human interactions by capturing human-human attention. Notably, self-attention can also be viewed as interchangeable with certain GNN implementations in social navigation, as it resembles a single-hop GAT operating on a fully connected graph.

2 TRAINING ENVIRONMENT

This section examines essential components of training environments for social navigation, including methods for collision-avoidance training, crowd behavior modeling, spatial layout designs, and methods for defining crowd start-goal configurations. These components are integrated within physics-based simulation frameworks, accurately replicating robot dynamics and sensory feedback to create realistic training settings.

2.1 Collision Avoidance Training

Collision avoidance behaviors are trained using various strategies based on environmental complexity and obstacle dynamics. Static collision avoidance is often trained with static obstacles like walls (103). Dynamic collision avoidance is trained with dynamic obstacles that move linearly, which may simulate self-collision (104), or ignore self-collision (105), or exhibit simple oscillatory motion along predefined paths (106). However, these typically assume non-interacting movement, limiting realism. Multi-agent RL (MARL) with centralized training and decentralized execution (CTDE) (107) enables robots to learn avoidance behaviors with shared experience but independent operation (108). While data-efficient, training against identical agents may not reflect human unpredictability. Training with simulated human crowds introduces non-linear, unpredictable dynamics; crowds may be cooperative or non-cooperative, with behaviors sampled uniformly (109), from Gaussian distributions (110), or a mixture of different crowd models (111) to enhance robustness.

2.2 Crowd Behavior Modeling

Crowd behavior methods are pivotal for training robots to navigate effectively among realistic crowds. These methods are generally classified into 3 categories: *Microscopic*, *Macroscopic*, and *Mesoscopic* (112). *Microscopic* methods focus on individual humans, providing detailed modeling of each human's behavior. *Macroscopic* methods (113) treat the crowd as a continuous flow, similar to fluids, focusing on overall movements over individual behaviors but lacking individual heterogeneous behaviors. *Mesoscopic* methods (114) combine elements of both microscopic individual agent behavior for local intra-group interactions and macroscopic principles for group-level dynamics.

Here, we focus on *Microscopic* methods, which are crucial for training social navigation policies due to their detailed individual-level interactions. We highlight force-based, velocity-based, data-driven, and optimization-based methods.

Force-based Models. Force-based models, initially proposed as the Social Force Model (SFM) (115, 116), simulate pedestrian behavior through attractive and repulsive forces based on Newton's second law: $F_i = f_i^d + \sum_o f_{io}^{obs} + \sum_j f_{ij}^{ped}$. The attractive force guides pedestrians toward their destinations f^d, while repulsive forces maintain personal space and avoid collisions with obstacles f^{obs} and other pedestrians f^{ped}. Variations include anticipatory approaches (117), visual perception models (118), group behavior extensions (119), and scenario-specific adaptations for bidirectional flows and bottlenecks (120, 121).

Velocity-based Models. Velocity-based models utilize velocity obstacles (VO) (42) and their extensions, reciprocal velocity obstacles (RVO) (43) and optimal reciprocal collision avoidance

(ORCA) (44). These methods compute collision-free velocities $VO^{\tau}_{A|B}$ by identifying velocity spaces that prevent collisions within a defined time horizon τ. ORCA refines RVO by optimizing pedestrians' velocities for efficiency and collision avoidance. Variants of ORCA include the collision-free speed model (CFSM) (122), which adjusts individual velocities to maintain safe speeds and prevent collisions, and Curtis and Manocha (123), which adapts pedestrian velocity preferences based on the surrounding crowd density.

Data-driven Models. Data-driven approaches utilize real-world crowd trajectory datasets to replicate or generalize crowd behaviors. Basic methods replay real crowd movements, while more advanced techniques involve optimizing parameters of existing models, such as force-based or velocity-based methods, to match real crowd behaviors (124, 125). Recent developments include integrating deep learning, such as the Deep SFM (126), which replaces traditional parameters with a neural network, enhancing realism by learning complex interactions directly from data.

Optimization-based Models. Optimization-based methods optimize a global objective function over all pedestrians, either over the forces (127), velocity obstacles (128), or energy-based models (129). For instance, PLEdestrians (130) operates on the principle of least effort (PLE), where pedestrians select trajectories that minimize expected effort. Similarly, Hoogendoorn and Bovy (131) optimizes a utility function that represents the expected cost of walking to the destination. The optimal steps model (OSM) (132) models motion as a sequence of discrete footstep events, where each footstep is explicitly optimized based on a distance field.

2.3 Map Design

Map design plays a vital role in developing and testing social navigation algorithms, enabling the simulation of diverse real-world environments, such as open areas, corridors, intersections, doorways, and urban landscapes.

Open spaces, such as public spaces where robots move freely with no predefined paths, present the robot with the task of navigating through areas with varying crowd densities. Open spaces may also include static obstacles, such as trash cans, tables, and benches, introducing additional challenges for navigation. *Corridors* are commonly used to evaluate navigation through narrow, constrained spaces. A typical scenario involves a straight corridor where the robot attempts to navigate around other humans moving in one or both directions. Variations may include adding obstacles, varying corridor width, or introducing curves and turns. *Intersections*, where multiple paths converge, offer a more complex environment for social navigation. These scenarios typically feature intersecting corridors in the form of cross or T-shapes, which require the robot to yield, merge, or cross paths with humans while avoiding collisions.

Doorways simulate the challenge of navigating through a narrow passage where multiple humans may compete for limited space, requiring the robot to wait, yield, or proceed. Complexity can be adjusted by varying the doorway width and the number of dynamic humans approaching from both directions. *Urban* environments present unique challenges for testing social navigation due to their complex and dynamic nature. These scenarios feature diverse pathways, such as sidewalks and crossings, where robots and humans must navigate around vehicles or follow traffic signals. Such settings evaluate a robot's ability to handle varied interactions and adapt to changing conditions. *Real-world* maps enable testing in realistic environments by incorporating publicly available 2D maps of apartments, offices, and malls (133, 134), as well as 3D maps (135, 136). These maps serve as the foundation for simulated settings, featuring complex layouts with diverse spatial configurations. This complexity provides a more authentic context for evaluating social navigation (137).

2.4 Crowd Goal Assignment

Crowd behavior algorithms define individuals' movement patterns as they navigate through

environments, but crowd dynamics require assigning each agent a specific start and goal location. Different methods, from scenario-based to data-driven, are used for goal assignment across various environments.

Scenario-based goal assignment involves defining humans' goals according to the scenario context. For instance, in an evacuation scenario, humans are assigned goals such as reaching the nearest exits. In a corridor scenario, human goals involve moving from one end of the corridor to the other. Starting points are typically random based on the desired crowd density or derived from empirical distributions, such as density distribution detailed by Corbetta et al. (138) for corridor flows.

Data-driven goal assignment utilizes real-world data to determine the start and goal locations, reflecting the movement patterns observed in real crowds (139).

Random goal assignment randomly assigns start and goal locations, usually following a uniform distribution within the environment. This approach is more suited for open spaces like parks or malls, where individuals move freely without a common objective.

3 SOCIAL NAVIGATION BENCHMARKING

This section outlines the benchmarking setup for evaluating state-of-the-art social navigation planners from various categories in realistic and challenging scenarios. We achieve consistent evaluation by adapting planners to handle static obstacles such as walls. The section covers evaluation metrics, testing scenarios, and planner configurations, detailing both baseline and learning-based planners, including their observation space, neural network architecture, and training parameters. We benchmark each planner to provide insights into their strengths, limitations, and real-world applicability.

3.1 Benchmark Setup

The benchmark setup consists of a circular robot with a 0.2 m radius, a maximum velocity of 1.5 m/s, and a maximum acceleration of 1.5 m/s^2, operating under holonomic dynamics. Humans are modeled as circles with a 0.3 m radius, a preferred velocity of 1.0 m/s with 0.1 standard deviation, a maximum velocity of 1.2 m/s, and a maximum acceleration of 2 m/s^2. A collision is defined when the robot's boundary comes within 0.01 m of a human or obstacle boundary.

Evaluation metrics include the human and obstacle distances, measured as the average minimum boundary-to-boundary distance between the robot and the nearest humans and obstacles per episode. The crowdness metric quantifies the average number of humans whose centers fall within a defined radius of the robot's boundary. The path-ratio metric is computed as the ratio of the total traveled distance to the Euclidean distance from start to goal, averaged over successful episodes. Each planner is tested for 1,000 episodes per scenario, with a maximum episode duration of 35 seconds, after which timeouts are recorded. The robot successfully reaches the goal if it arrives within 0.3 m of the target. A seed value of 42 is used for all random processes to ensure reproducibility, and the reported 95% confidence interval is computed using the z-score.

3.2 Benchmark Scenarios

Benchmark scenarios are selected to cover the majority of challenges a robot may encounter when navigating human-crowded indoor and outdoor environments. These scenarios aim to simulate diverse real-world navigation contexts, ensuring the benchmark evaluates planners comprehensively across critical aspects of social navigation (140, 141, 111). The robot's start and goal positions, along with the environment's dimensions and crowdedness, are uniformly and randomly determined within predefined bounds. To avoid infeasible or unsafe configurations, the robot's starting position is defined with a safety radius that

maintains an appropriate distance from humans or obstacles.

In each scenario, the start and goal positions are randomly sampled within predefined map regions, while ensuring the start-goal distance falls within a specified range. To maintain feasibility, the robot's initial position is placed at least 0.5 m from humans and 0.15 m from obstacles. Scenario parameters, including spatial dimension and crowd size, are uniformly sampled within predefined ranges, as detailed in Table 1.

Static Scenario. In the static scenario, the focus is on human-free environments with static obstacles, testing the robot's ability to navigate efficiently and smoothly in narrow, confined spaces such as offices, warehouses, or retail shops. The map used for this scenario is an L-shaped environment with six walls that form sharp turns and narrow paths. It consists of a horizontal section three times its width and a vertical section four times its width, with a 50% probability of being flipped vertically.

Doorway Scenario. The doorway scenario presents a common real-world challenge where robots and humans encounter each other at chokepoints. This scenario evaluates the robot's behavior in yielding, passing, or maintaining safe distances in situations with constrained passage (142, 143). The doorway map consists of two square rooms connected by a central doorway. Humans in the environment attempt to move in the opposite direction of the robot, creating natural encounters. During evaluation, the robot spawns in one room with equal probability, and the goal is set in the other.

Corridor Scenario. Corridor scenarios mimic indoor environments such as corridors in airports, shopping malls, or office buildings. These narrow spaces provide limited overtaking opportunities and test the robot's ability to integrate into crowd flows without disrupting them (111). Crowd movements are either single-directional or bidirectional. In bidirectional flows, a soft separator is present, but humans occasionally cross it. Humans are respawned upon reaching the corridor's end to maintain continuous flow. The robot's navigation may involve traveling to a random point or moving along the length of the corridor. The scenario features crowd flow that is unidirectional in half of the episodes and bidirectional in the rest.

Intersection Scenario. Intersections present environments where two corridors meet, introducing complex navigation challenges even for human pedestrians. These scenarios assess the robot's ability to predict human trajectories, yield when appropriate, and navigate efficiently through areas of high interaction density (141). The intersection map is based on the corridor scenario, with two perpendicular corridors forming a cross. The crowd flow is unidirectional in half of the episodes.

Open Space Scenario. Open space scenarios simulate environments such as public squares, open office areas, or parks where movement is less constrained, with two possible crowd setups. In the random setup, human agents have uniformly random start and goal points, with at most half their paths intersecting with the robot's trajectory (111). This configuration creates diverse interaction patterns and collision risks. In the data-driven setup, human behavior is modeled using real-world data from the ETH (144) and UCY (139) datasets, which capture realistic pedestrian movements. Model-based crowd behavior (ORCA) enables humans to react dynamically to the robot's presence. The open space random scenario increases complexity by ensuring that 75% of humans cross the robot's direct start-to-goal path, while the open space data-driven scenario sets the crowd size based on real-world data, allowing up to 40 individuals to be present simultaneously.

3.3 Benchmark Planners

For each planner, we define the observation space, neural network architecture, and training process. Baseline planners use the full state space as their observations, while learning-based planners receive the robot state, LiDAR readings quantized to 40 rays, and the positions of up to 10 nearest humans. The robot state includes its velocity in

Scenario	Parameter	Min.	Max.
All	Robot Goal Distance	15	25
Static	Corridor Width	1	4
Static	Robot Goal Distance	3	25
Doorway	Robot Goal Distance	3	25
Doorway	Crowd Size	0	4
Doorway	Room Width	4	6
Doorway	Door Width	0.8	4
Corridor	Crowd Size	20	40
Corridor	Corridor Width	4.5	7
Intersection	Crowd Size	30	40
Intersection	Corridor Width	3.5	7
Openspace Random	Crowd Size	30	40

Table 1. Uniformly sampled scenario parameters with their minimum and maximum values.

the x- and y-axes and its relative distance to the goal in both axes. Most learning-based planners (except Imitation Learning) were trained using the PPO algorithm from the RL-Games library (145). Shared hyperparameters include a discount factor of $\gamma = 0.99$, a learning rate of 3×10^{-4}, a gradient norm of 1.5, a clip parameter of 0.2, an epoch size of 300, and 256 parallel environments, with most values optimized via Optuna (146). Training used the Adam optimizer with an adaptive learning rate scheduler, which dynamically adjusted the learning rate based on KL divergence, decreasing it if KL exceeded twice a threshold, increasing it if KL fell below half, and maintaining it when within bounds.

The reward function includes the following components: goal reaching ($+200$), timeout penalty (-50), collision penalty (-200), human distance cost ($+2$) with an activation threshold of 0.5 m, and goal progress difference ($+10$).

Baseline Planners. The baseline planners consist of Python-based implementations of ORCA (147), SFM (148), and DWA (149). The DWA implementation was modified to incorporate constant-velocity projections for moving obstacles. Since ORCA and SFM are simpler reactive planners, they operate at 100 Hz, whereas DWA and learning-based planners run at 20 Hz.

End-to-End Planner. The end-to-end planner processes LiDAR readings using two 1D-CNN layers with a kernel size of 3, a stride of 1, and 32 channels. The CNN output is passed through a fully connected (FC) layer of size 256, concatenated with the robot state, and then processed by a single GRU layer of size 256 with leaky ReLU activation. The final layer consists of an action output, which uses a tanh activation to bound values within $[-1, 1]$, and a value network without an activation function. This final layer structure is shared across all learning-based planners. Training was performed using PPO with a horizon length of 512, a batch size of 8192, and a sequence length of 32 for the GRU to maintain temporal context.

Imitation Learning-based Planner. The imitation learning-based planner is trained on a dataset of 35,000 successful episodes generated by the human attention-based planner. Training follows the Behavioral Cloning (BC) algorithm from the Imitation library (150), using an Adam optimizer, a learning rate of 10^{-3}, and an epoch size of 5. The network architecture is based on the human attention-based planner.

Human Position-based Planner. The human position-based planner is built on GA3C-CADRL (151), utilizing an actor-critic policy with an LSTM layer to encode human states, which consist of each human's position and velocity relative to the robot. The LiDAR input is processed through two 1D-CNN layers with kernel sizes of 5 and 3, a stride of 2, and 32 channels, followed by an FC layer of size 128. The LSTM layer, with a hidden size of 64, encodes all human states. The outputs of the LSTM and LiDAR network are concatenated and passed through two FC layers of size 256. Training was conducted using PPO with a horizon length of 512 and a batch size of 8192.

Human Attention-based Planner. The human attention-based planner builds on SARL (110), an attention-based network designed to model attentions between the robot and humans. We

Table 2. Static Scenario Experiment

Planner	Success	Collision	Timeout	Running Time (s)	Path Ratio	Avg. Vel. (m/s)	Avg. Accel. (m/s^2)	Min. Obs. Dist. (m)
SFM	75.3%	6.0%	18.7%	5.89±0.16	1.18±0	0.620±0	0.572±0	**0.285±0**
ORCA	**99.9%**	**0.0%**	**0.1%**	8.91±0.19	**1.10±0**	0.720±0	**0.368±0**	0.173±0
DWA	95.4%	4.6%	0.0%	5.53±0.15	1.11±0	1.236±0	1.012±0	0.224±0
End-to-End	94.6%	0.2%	5.2%	5.76±0.16	1.14±0	0.888±0	0.613±0	0.238±0
Imitation Learning	17.1%	20.0%	62.9%	12.11±1.2	1.40±0	0.428±0	1.828±0	0.131±0
Human-Pose	93.4%	4.0%	2.6%	5.26±0.15	1.14±0	1.148±0	1.074±0	0.218±0
Human-Interaction	91.5%	1.3%	7.2%	5.39±0.16	1.14±0	0.883±0	0.850±0	**0.285±0**
Prediction Planner	**99.9%**	**0.0%**	**0.1%**	**4.97±0.13**	1.13±0	**1.336±0**	0.960±0	0.242±0
Safe Planner	91.0%	1.1%	7.9%	5.31±0.16	1.13±0	0.844±0	0.894±0	0.258±0

Table 3. Doorway Scenario Experiment

Planner	Success	Collision	Timeout	Running Time (s)	Path Ratio	Avg. Vel. (m/s)	Avg. Accel. (m/s^2)	Min. Obs. Dist. (m)	Min. Human Distance (m)	Crowdness (1m Radius)
SFM	47.2%	39.2%	13.6%	6.07±0.28	1.22±0	0.536±0	0.971±0	0.393±0	0.183±0	1.023±0
ORCA	47.3%	45.3%	7.4%	7.75±0.26	**1.05±0**	0.515±0	**0.474±0**	**0.426±0**	0.167±0	0.770±0
DWA	64.0%	17.3%	18.7%	**5.72±0.20**	1.11±0	0.473±0	0.897±0	0.264±0	0.184±0	0.969±0
End-to-End	81.7%	4.3%	14.0%	7.73±0.27	1.24±0	0.521±0	0.713±0	0.275±0	0.283±0	**0.562±0**
Imitation Learning	46.4%	23.0%	30.6%	6.56±0.35	1.27±0	0.518±0	1.843±0	0.121±0	0.262±0	0.814±0
Human-Pose	85.7%	10.9%	3.4%	6.76±0.16	1.30±0	0.877±0	1.434±0	0.245±0	0.245±0	0.755±0
Human-Interaction	83.0%	14.1%	2.9%	5.93±0.16	1.19±0	0.916±0	1.259±0	0.158±0	0.233±0	0.852±0
Prediction Planner	**93.2%**	**3.2%**	3.6%	5.79±0.14	1.28±0	**0.993±0**	1.367±0	0.215±0	**0.310±0**	0.649±0
Safe Planner	84.4%	13.0%	**2.6%**	6.06±0.16	1.21±0	0.922±0	1.284±0	0.144±0	0.226±0	0.877±0

extend the original value-based model to an actor-critic framework and integrate a LiDAR processing network. The LiDAR network consists of two 1D-CNN layers with kernel sizes of 5 and 3, a stride of 2, and 32 channels, followed by an FC layer of size 128. Human states are processed through three FC layers of sizes 150, 100, and 50 to generate an initial embedding. This embedding is combined with the global state, defined as the mean of all human embeddings. An attention mechanism (FC $100 \rightarrow 1$) followed by a softmax function, computes attention scores, which are applied to refine the final human embedding. If no humans are detected, a learned embedding is used instead. The LiDAR and human embeddings are concatenated and passed through two FC layers of size 256. The model was trained using PPO with a horizon length of 512 and a batch size of 4096.

Human Prediction-based Planner. The prediction-based planner extends the RGL planner (152), incorporating the robot state and LiDAR input to predict human trajectories in the robot frame. A predicted 5-step human trajectory is then processed by an actor-critic policy inspired by SARL (110), which is designed to handle multi-step human motion. When no humans are present, a learned embedding is used to pad the human embedding output. The LiDAR network comprises two 1D-CNN layers with kernel sizes of 5 and 3, a stride of 2, and 32 channels, followed by an FC layer of size 128. The predictor network first applies an FC layer to encode the robot and human states into a 64-dimensional embedding, forming a fully connected graph of the robot and humans. This is then processed through two graph convolutional network (GCN) layers with skip connections and an embedded Gaussian similarity function. The LiDAR output is concatenated with the GCN-derived human embedding and passed through an FC layer of size 64, followed by a final output layer of size 4 to predict the next human state. The predictor network is pre-trained using data collected from the end-to-end planner, remains frozen during

Table 4. Corridor Scenario Experiment

Planner	Success	Collision	Timeout	Running Time (s)	Path Ratio	Avg. Vel. (m/s)	Avg. Accel. (m/s^2)	Min. Obs. Dist. (m)	Min. Human Distance (m)	Crowdness (1m Radius)
SFM	82.1%	14.3%	3.6%	**13.80±0.13**	**1.0±0**	1.346±0	**0.292±0**	**1.179±0**	0.187±0	1.402±0
ORCA	90.8%	9.1%	0.1%	15.52±0.18	1.01±0	1.268±0	0.591±0	1.132±0	0.226±0	1.556±0
DWA	**94.8%**	5.2%	**0.0%**	14.73±0.14	1.01±0	**1.357±0**	0.790±0	0.838±0	**0.363±0**	1.374±0
End-to-End	79.2%	19.5%	1.3%	15.32±0.22	1.04±0	1.297±0	0.879±0	0.928±0	0.308±0	**1.169±0**
Imitation Learning	94.2%	3.9%	1.9%	15.60±0.19	1.04±0	1.290±0	1.779±0	0.705±0	0.284±0	1.342±0
Human-Pose	87.2%	6.0%	6.8%	16.26±0.25	1.08±0	1.271±0	1.249±0	0.895±0	0.324±0	1.315±0
Human-Interaction	93.8%	4.3%	1.9%	14.92±0.18	1.03±0	1.317±0	0.936±0	0.732±0	0.276±0	1.426±0
Prediction Planner	92.0%	6.2%	1.8%	14.79±0.18	1.03±0	1.336±0	0.978±0	0.888±0	0.299±0	1.381±0
Safe Planner	94.5%	**3.3%**	2.2%	14.94±0.18	1.03±0	1.316±0	0.947±0	0.720±0	0.278±0	1.448±0

Table 5. Intersection Scenario Experiment

Planner	Success	Collision	Timeout	Running Time (s)	Path Ratio	Avg. Vel. (m/s)	Avg. Accel. (m/s^2)	Min. Obs. Dist. (m)	Min. Human Distance (m)	Crowdness (1m Radius)
SFM	60.7%	37.2%	2.1%	**13.77±0.16**	**1.004±0**	**1.365±0**	**0.333±0**	**1.068±0.04**	0.147±0	1.154±0
ORCA	61.8%	37.4%	**0.8%**	15.50±0.22	1.007±0	1.276±0	0.515±0	1.055±0.04	0.161±0	1.343±0
DWA	85.8%	13.2%	1.0%	14.88±0.15	1.030±0	1.351±0	0.875±0	0.733±0.04	0.258±0	1.173±0
End-to-End	79.9%	18.8%	1.3%	16.17±0.22	1.067±0	1.268±0	1.069±0	0.816±0.03	0.208±0	1.126±0
Imitation Learning	88.6%	8.0%	3.4%	16.70±0.24	1.079±0	1.225±0	1.807±0	0.543±0.03	0.233±0	1.229±0
Human-Pose	81.8%	7.4%	10.8%	17.86±0.31	1.163±0	1.228±0	1.411±0	0.759±0.03	0.282±0	**1.048±0**
Human-Interaction	92.5%	3.8%	3.7%	16.20±0.22	1.072±0	1.248±0	1.143±0	0.611±0.03	0.236±0	1.213±0
Prediction Planner	92.8%	5.6%	1.6%	16.24±0.22	1.090±0	1.291±0	1.199±0	0.797±0.03	**0.290±0**	1.111±0
Safe Planner	**94.4%**	**3.1%**	2.5%	15.90±0.21	1.068±0	1.278±0	1.120±0	0.642±0.03	0.241±0	1.184±0

planner training, and was trained for 40 epochs with the Adam optimizer (learning rate 10^{-3}) using MSE loss. The policy was trained using PPO with a horizon length of 512 and a batch size of 4096.

Safety-aware Planner. The safety-aware planner, inspired by Linh et al. (153), combines multiple policies with a policy switcher for adaptive behavior. It integrates the ORCA planner (43) and the human attention-based planner (110). The policy switcher dynamically selects a planner based on obstacle proximity, favoring the learning-based planner when obstacles are within 3 m and switching to the model-based planner otherwise.

3.4 Results

Across six benchmark scenarios, learning-based planners consistently outperform model-based methods in terms of success rate and safety, with detailed results summarized in Tables 2–7. In the static scenario (Table 2), all methods achieve low collision rates; however, learning-based planners generally complete tasks faster, whereas model-based methods like ORCA favor higher success at the expense of longer run times. In the Doorway scenario (Table 3), where human-robot interactions are more frequent, learning-based planners demonstrate better adaptability, resulting in safer navigation. Both planner types perform similarly in the corridor scenario (Table 4), but learning-based methods, particularly prediction-based approaches, excel in the intersection (Table 5) and in open space scenario (Tables 6 and 7), where they navigate dynamic environments more effectively. Within the learning-based group, end-to-end planner stands out for safety, while the Human position-based and Human attention-based planners excel in open and crowded environments, respectively, and the Prediction-based planner achieves the highest overall performance.

3.4.1 Computational Cost Analysis

The computational time of each planner (running on a desktop with Intel Core i7-13700F and NVIDIA RTX 4080), as shown in Table 8, reflects both the complexity of its underlying architecture and the influence of the crowd size. Model-based methods like ORCA rely on

Table 6. Openspace Random Scenario Experiment

Planner	Success	Collision	Timeout	Running Time (s)	Path Ratio	Avg. Vel. (m/s)	Avg. Accel. (m/s^2)	Min. Human Distance (m)	Crowdness (1m Radius)
SFM	33.1%	66.4%	0.5%	11.51±0.1	**1.01±0**	1.329±0	**0.519±0**	0.094±0	1.813±0
ORCA	18.3%	81.1%	0.6%	13.47±0.3	1.03±0	1.234±0	0.702±0	0.098±0	1.909±0
DWA	38.4%	61.6%	**0.0%**	**10.22±0.1**	1.06±0	**1.665±0**	1.088±0	0.133±0	1.419±0
End-to-End	67.1%	30.3%	2.6%	19.45±0.4	1.38±0	1.114±0	1.264±0	0.216±0	0.783±0
Imitation Learning	93.8%	5.4%	0.8%	16.58±0.3	1.27±0	1.210±0	1.862±0	0.193±0	1.156±0
Human-Pose	**95.7%**	**4.1%**	0.2%	17.64±0.2	1.55±0	1.392±0	1.555±0	**0.356±0**	**0.554±0**
Human-Interaction	94.7%	5.2%	0.1%	15.39±0.2	1.25±0	1.286±0	1.500±0	0.193±0	1.201±0
Prediction Planner	93.4%	6.1%	0.5%	17.88±0.3	1.38±0	1.218±0	1.531±0	0.292±0	0.819±0
Safe Planner	94.5%	5.5%	**0.0%**	15.45±0.2	1.25±0	1.285±0	1.475±0	0.196±0	1.177±0

Table 7. Openspace Data-driven Scenario Experiment

Planner	Success	Collision	Timeout	Running Time (s)	Path Ratio	Avg. Vel. (m/s)	Avg. Accel. (m/s^2)	Min. Human Distance (m)	Crowdness (1m Radius)
SFM	63.6%	36.4%	**0.0%**	**10.05±0.1**	**1.003±0**	1.381±0	**0.382±0**	0.390±0.05	1.067±0
ORCA	50.9%	48.7%	0.4%	11.75±0.3	1.012±0	1.231±0	0.491±0	0.453±0.06	1.301±0
DWA	67.1%	32.9%	**0.0%**	10.08±0.2	1.058±0	**1.504±0**	1.007±0	0.405±0.04	1.325±0
End-to-End	92.2%	7.6%	0.2%	14.07±0.3	1.191±0	1.200±0	1.017±0	0.655±0.04	**0.399±0**
Imitation Learning	96.0%	2.4%	1.6%	13.92±0.3	1.188±0	1.190±0	1.820±0	0.393±0.03	0.776±0
Human-Pose	**98.2%**	**1.8%**	**0.0%**	11.85±0.2	1.236±0	1.487±0	1.338±0	**0.703±0.04**	0.356±0
Human-Interaction	98.1%	1.9%	**0.0%**	11.14±0.2	1.128±0	1.438±0	1.221±0	0.398±0.03	0.847±0
Prediction Planner	97.5%	2.5%	**0.0%**	12.01±0.2	1.186±0	1.402±0	1.262±0	0.660±0.04	0.426±0
Safe Planner	97.0%	3.0%	**0.0%**	13.20±0.2	1.204±0	1.298±0	1.334±0	0.241±0.01	1.252±0

Planner	No Humans	1 Human	5 Humans	10 Humans
SFM	1.800±0.0	2.349±0.01	2.385±0.01	2.866±0.01
ORCA	0.720±0.0	0.823±0.01	0.728±0.01	0.795±0.0
DWA	2.409±0.02	7.095±0.03	6.292±0.05	9.710±0.06
End-to-End	0.518±0.0	0.521±0.00	0.529±0.0	0.526±0.0
Human-Pose	1.164±0.0	1.172±0.00	1.163±0.0	1.162±0.0
Human-Interaction	0.707±0.0	0.715±0.00	0.711±0.0	0.710±0.0
Prediction Planner	2.328±0.01	2.329±0.01	2.334±0.01	2.335±0.0
Safe Planner	0.720±0.0	0.745±0.02	0.752±0.0	0.752±0.0

Table 8. Computational time (ms) comparison of planners with varying crowd sizes.

efficient implementation for improved execution times, while methods like SFM and DWA exhibit longer computation times, particularly in crowded scenarios. In contrast, learning-based planners exhibit greater stability in execution times, benefiting from GPUs for parallel processing. This allows them to maintain consistently low execution times, even as the number of humans in the environment increases. Prediction-based planner, while slightly more computationally intensive, remains steady across all cases. These results highlight the trade-off between architectural complexity and scalability in dynamic environments, where learned policies tend to offer more predictable and efficient execution times compared to some model-based approaches. The execution times of the Imitation Learning planner depends on the specific architecture used; in our implementation, it shares a similar structure with the Human attention-based planner, resulting in comparable times.

With the exception of the end-to-end planner, most tested planners rely on human detection and tracking, which significantly increases their total runtime. Real-time object detection models like YOLO balance speed and accuracy, with inference times typically ranging from a few to tens of milliseconds per frame, depending on the model variant and hardware configuration (154, 155). Additionally, tracking methods like SORT (156) offer efficient and lightweight tracking with minimal computational overhead, making them ideal for real-time applications. SORT operates

within a few milliseconds per frame, depending on the hardware configuration (157).

REFERENCES

1 .Cadena C, Carlone L, Carrillo H, Latif Y, Scaramuzza D, Neira J, et al. Past, present, and future of simultaneous localization and mapping: Toward the robust-perception age. *IEEE Transactions on robotics* **32** (2016) 1309–1332.

2 .Montemerlo M, Thrun S, Koller D, Wegbreit B, et al. Fastslam: A factored solution to the simultaneous localization and mapping problem. *Aaai/iaai* **593598** (2002).

3 .Campos C, Elvira R, Rodríguez JJG, Montiel JM, Tardós JD. Orb-slam3: An accurate open-source library for visual, visual–inertial, and multimap slam. *IEEE Transactions on Robotics* **37** (2021) 1874–1890.

4 .Czarnowski J, Laidlow T, Clark R, Davison AJ. Deepfactors: Real-time probabilistic dense monocular slam. *IEEE Robotics and Automation Letters* **5** (2020) 721–728.

5 .Placed JA, Strader J, Carrillo H, Atanasov N, Indelman V, Carlone L, et al. A survey on active simultaneous localization and mapping: State of the art and new frontiers. *IEEE Transactions on Robotics* (2023).

6 .Dijkstra EW. A note on two problems in connexion with graphs. *Numerische mathematik* **1** (1959) 269–271.

7 .Nilsson NJ. *Principles of artificial intelligence* (: Springer Science & Business Media) (1982).

8 .Dolgov D, Thrun S, Montemerlo M, Diebel J. Path planning for autonomous vehicles in unknown semi-structured environments. *The international journal of robotics research* **29** (2010) 485–501.

9 .Stentz A, Mellon IC. Optimal and efficient path planning for unknown and dynamic environments. *International Journal of Robotics and Automation* **10** (1995) 89–100.

10 .Lozano-Pérez T, Wesley MA. An algorithm for planning collision-free paths among polyhedral obstacles. *Communications of the ACM* **22** (1979) 560–570.

11 .Aurenhammer F. Voronoi diagrams—a survey of a fundamental geometric data structure. *ACM Computing Surveys (CSUR)* **23** (1991) 345–405.

12 .Kallman M, Mataric M. Motion planning using dynamic roadmaps. *IEEE International Conference on Robotics and Automation, 2004. Proceedings. ICRA'04. 2004* (IEEE) (2004), vol. 5, 4399–4404.

13 .Švestka P, Overmars MH. Motion planning for carlike robots using a probabilistic learning approach. *The International Journal of Robotics Research* **16** (1997) 119–143.

14 .LaValle S. Rapidly-exploring random trees: A new tool for path planning. *Research Report 9811* (1998).

15 .Karaman S, Frazzoli E. Sampling-based algorithms for optimal motion planning. *The international journal of robotics research* **30** (2011) 846–894.

16 .Otte M, Frazzoli E. Rrtˆ x rrt x: Real-time motion planning/replanning for environments with unpredictable obstacles. *Algorithmic foundations of robotics XI: selected contributions of the eleventh international workshop on the algorithmic foundations of robotics* (Springer) (2015), 461–478.

17 .Naderi K, Rajamäki J, Hämäläinen P. Rt-rrt* a real-time path planning algorithm based on rrt. *Proceedings of the 8th ACM SIGGRAPH Conference on Motion in Games* (2015), 113–118.

18 .Bency MJ, Qureshi AH, Yip MC. Neural path planning: Fixed time, near-optimal path generation via oracle imitation. *2019 IEEE/RSJ International Conference on Intelligent Robots and Systems (IROS)* (IEEE) (2019), 3965–3972.

19 .Palmieri L, Arras KO. Distance metric learning for rrt-based motion planning with constant-time inference. *2015 IEEE International conference on robotics and automation (ICRA)* (IEEE) (2015), 637–643.

20 .Chiang HTL, Hsu J, Fiser M, Tapia L, Faust A. Rl-rrt: Kinodynamic motion planning via learning reachability estimators from rl policies. *IEEE Robotics and Automation Letters* **4** (2019) 4298–4305.

21 .Wang J, Chi W, Li C, Wang C, Meng MQH. Neural rrt*: Learning-based optimal path planning. *IEEE Transactions on Automation Science and Engineering* **17** (2020) 1748–1758.

22 .Qureshi AH, Miao Y, Simeonov A, Yip MC. Motion planning networks: Bridging the gap between learning-based and classical motion planners. *IEEE Transactions on Robotics* **37** (2020) 48–66.

23 .Yonetani R, Taniai T, Barekatain M, Nishimura M, Kanezaki A. Path planning using neural a* search. *International conference on machine learning* (PMLR) (2021), 12029–12039.

24 .Fox D, Burgard W, Thrun S. The dynamic window approach to collision avoidance. *IEEE Robotics & Automation Magazine* **4** (1997) 23–33.

25 .Faust A, Oslund K, Ramirez O, Francis A, Tapia L, Fiser M, et al. Prm-rl: Long-range robotic navigation tasks by combining

reinforcement learning and sampling-based planning. *2018 IEEE international conference on robotics and automation (ICRA)* (IEEE) (2018), 5113–5120.

26. Gao J, Ye W, Guo J, Li Z. Deep reinforcement learning for indoor mobile robot path planning. *Sensors* **20** (2020) 5493.

27. Kästner L, Zhao X, Buiyan T, Li J, Shen Z, Lambrecht J, et al. Connecting deep-reinforcement-learning-based obstacle avoidance with conventional global planners using waypoint generators. *2021 IEEE/RSJ International Conference on Intelligent Robots and Systems (IROS)* (IEEE) (2021), 1213–1220.

28. Lu DV, Hershberger D, Smart WD. Layered costmaps for context-sensitive navigation. *2014 IEEE/RSJ International Conference on Intelligent Robots and Systems* (IEEE) (2014), 709–715.

29. Guo H, Meng Z, Huang Z, Kang LW, Chen Z, Meghjani M, et al. Safe path planning with gaussian process regulated risk map. *2019 IEEE/RSJ International Conference on Intelligent Robots and Systems (IROS)* (IEEE) (2019), 2044–2051.

30. Kollmitz M, Hsiao K, Gaa J, Burgard W. Time dependent planning on a layered social cost map for human-aware robot navigation. *2015 European Conference on Mobile Robots (ECMR)* (IEEE) (2015), 1–6.

31. Fang F, Shi M, Qian K, Zhou B, Gan Y. A human-aware navigation method for social robot based on multi-layer cost map. *International Journal of Intelligent Robotics and Applications* **4** (2020) 308–318.

32. Dugas D, Cai K, Andersson O, Lawrance N, Siegwart R, Chung JJ. Flowbot: Flow-based modeling for robot navigation. *2022 IEEE/RSJ International Conference on Intelligent Robots and Systems (IROS)* (IEEE) (2022), 8799–8805.

33. Jaillet L, Cortés J, Siméon T. Sampling-based path planning on configuration-space costmaps. *IEEE Transactions on Robotics* **26** (2010) 635–646.

34. Mainprice J, Sisbot EA, Jaillet L, Cortés J, Alami R, Siméon T. Planning human-aware motions using a sampling-based costmap planner. *2011 IEEE International Conference on Robotics and Automation* (IEEE) (2011), 5012–5017.

35. Sánchez-Ibáñez JR, Pérez-del Pulgar CJ, García-Cerezo A. Path planning for autonomous mobile robots: A review. *Sensors* **21** (2021) 7898.

36. Khatib O. Real-time obstacle avoidance for manipulators and mobile robots. *The international journal of robotics research* **5** (1986) 90–98.

37. Qixin C, Yanwen H, Jingliang Z. An evolutionary artificial potential field algorithm for dynamic path planning of mobile robot. *2006 IEEE/RSJ International Conference on Intelligent Robots and Systems* (IEEE) (2006), 3331–3336.

38. Koren Y, Borenstein J, et al. Potential field methods and their inherent limitations for mobile robot navigation. *Icra* (1991), vol. 2, 1398–1404.

39. Borenstein J, Koren Y, et al. The vector field histogram-fast obstacle avoidance for mobile robots. *IEEE transactions on robotics and automation* **7** (1991) 278–288.

40. Ulrich I, Borenstein J. Vfh+: Reliable obstacle avoidance for fast mobile robots. *Proceedings. 1998 IEEE international conference on robotics and automation (Cat. No. 98CH36146)* (IEEE) (1998), vol. 2, 1572–1577.

41. Ulrich I, Borenstein J. Vfh/sup*: Local obstacle avoidance with look-ahead verification. *Proceedings 2000 ICRA. Millennium Conference. IEEE International Conference on Robotics and Automation. Symposia Proceedings (Cat. No. 00CH37065)* (IEEE) (2000), vol. 3, 2505–2511.

42. Fiorini P, Shiller Z. Motion planning in dynamic environments using velocity obstacles. *The international journal of robotics research* **17** (1998) 760–772.

43. Van den Berg J, Lin M, Manocha D. Reciprocal velocity obstacles for real-time multi-agent navigation. *2008 IEEE international conference on robotics and automation* (Ieee) (2008), 1928–1935.

44. Van Den Berg J, Guy SJ, Lin M, Manocha D. Reciprocal n-body collision avoidance. *Robotics Research: The 14th International Symposium ISRR* (Springer) (2011), 3–19.

45. Brock O, Khatib O. High-speed navigation using the global dynamic window approach. *Proceedings 1999 ieee international conference on robotics and automation (Cat. No. 99CH36288C)* (IEEE) (1999), vol. 1, 341–346.

46. Seder M, Petrovic I. Dynamic window based approach to mobile robot motion control in the presence of moving obstacles. *Proceedings 2007 IEEE International Conference on Robotics and Automation* (IEEE) (2007), 1986–1991.

47. Quinlan S, Khatib O. Elastic bands: Connecting path planning and control. *[1993] Proceedings IEEE International Conference on Robotics and Automation* (IEEE) (1993), 802–807.

48 .Rösmann C, Hoffmann F, Bertram T. Timed-elastic-bands for time-optimal point-to-point nonlinear model predictive control. *2015 european control conference (ECC)* (IEEE) (2015), 3352–3357.

49 .Tuncer A, Yildirim M. Dynamic path planning of mobile robots with improved genetic algorithm. *Computers & Electrical Engineering* **38** (2012) 1564–1572.

50 .Lamini C, Benhlima S, Elbekri A. Genetic algorithm based approach for autonomous mobile robot path planning. *Procedia Computer Science* **127** (2018) 180–189.

51 .Nazarahari M, Khanmirza E, Doostie S. Multi-objective multi-robot path planning in continuous environment using an enhanced genetic algorithm. *Expert Systems with Applications* **115** (2019) 106–120.

52 .Yan Y, Li Y. Mobile robot autonomous path planning based on fuzzy logic and filter smoothing in dynamic environment. *2016 12th World congress on intelligent control and automation (WCICA)* (IEEE) (2016), 1479–1484.

53 .Song Q, Zhao Q, Wang S, Liu Q, Chen X. Dynamic path planning for unmanned vehicles based on fuzzy logic and improved ant colony optimization. *IEEE Access* **8** (2020) 62107–62115.

54 .Kapturowski S, Ostrovski G, Quan J, Munos R, Dabney W. Recurrent experience replay in distributed reinforcement learning. *International conference on learning representations* (2018).

55 .Sutton RS, Barto AG. *Reinforcement learning: An introduction* (: MIT press) (2018).

56 .Mnih V, Kavukcuoglu K, Silver D, Graves A, Antonoglou I, Wierstra D, et al. Playing atari with deep reinforcement learning. *arXiv preprint arXiv:1312.5602* (2013).

57 .Hasselt H. Double q-learning. *Advances in neural information processing systems* **23** (2010).

58 .Van Hasselt H, Guez A, Silver D. Deep reinforcement learning with double q-learning. *Proceedings of the AAAI conference on artificial intelligence* (2016), vol. 30.

59 .Bellemare MG, Dabney W, Munos R. A distributional perspective on reinforcement learning. *International conference on machine learning* (PMLR) (2017), 449–458.

60 .Williams RJ. Simple statistical gradient-following algorithms for connectionist reinforcement learning. *Machine learning* **8** (1992) 229–256.

61 .Mnih V, Badia AP, Mirza M, Graves A, Lillicrap T, Harley T, et al. Asynchronous methods for deep reinforcement learning. *International conference on machine learning* (PMLR) (2016), 1928–1937.

62 .Schulman J, Levine S, Abbeel P, Jordan M, Moritz P. Trust region policy optimization. *International conference on machine learning* (PMLR) (2015), 1889–1897.

63 .Kullback S, Leibler RA. On information and sufficiency. *The annals of mathematical statistics* **22** (1951) 79–86.

64 .Schulman J, Wolski F, Dhariwal P, Radford A, Klimov O. Proximal policy optimization algorithms. *arXiv preprint arXiv:1707.06347* (2017).

65 .Lillicrap TP, Hunt JJ, Pritzel A, Heess N, Erez T, Tassa Y, et al. Continuous control with deep reinforcement learning. *arXiv preprint arXiv:1509.02971* (2015).

66 .Fujimoto S, Hoof H, Meger D. Addressing function approximation error in actor-critic methods. *International conference on machine learning* (PMLR) (2018), 1587–1596.

67 .Haarnoja T, Zhou A, Abbeel P, Levine S. Soft actor-critic: Off-policy maximum entropy deep reinforcement learning with a stochastic actor. *International conference on machine learning* (PMLR) (2018), 1861–1870.

68 .Kingma DP, Welling M. Auto-encoding variational bayes. *arXiv preprint arXiv:1312.6114* (2013).

69 .Clavera I, Rothfuss J, Schulman J, Fujita Y, Asfour T, Abbeel P. Model-based reinforcement learning via meta-policy optimization. *Conference on Robot Learning* (PMLR) (2018), 617–629.

70 .Silver D, Hubert T, Schrittwieser J, Antonoglou I, Lai M, Guez A, et al. Mastering chess and shogi by self-play with a general reinforcement learning algorithm. *arXiv preprint arXiv:1712.01815* (2017).

71 .Kouvaritakis B, Cannon M. Model predictive control. *Switzerland: Springer International Publishing* **38** (2016) 13–56.

72 .Browne CB, Powley E, Whitehouse D, Lucas SM, Cowling PI, Rohlfshagen P, et al. A survey of monte carlo tree search methods. *IEEE Transactions on Computational Intelligence and AI in games* **4** (2012) 1–43.

73 .Kocsis L, Szepesvári C. Bandit based monte-carlo planning. *European conference on machine learning* (Springer) (2006), 282–293.

74 .Mattingley J, Wang Y, Boyd S. Receding horizon control. *IEEE Control Systems Magazine* **31** (2011) 52–65.

75 .Plaat A, Kosters W, Preuss M. Deep model-based reinforcement learning for high-dimensional problems, a survey. *arXiv preprint arXiv:2008.05598* (2020).

76. Silver D, Hasselt H, Hessel M, Schaul T, Guez A, Harley T, et al. The predictron: End-to-end learning and planning. *International Conference on Machine Learning* (PMLR) (2017), 3191–3199.

77. Oh J, Singh S, Lee H. Value prediction network. *Advances in neural information processing systems* **30** (2017).

78. Schrittwieser J, Antonoglou I, Hubert T, Simonyan K, Sifre L, Schmitt S, et al. Mastering atari, go, chess and shogi by planning with a learned model. *Nature* **588** (2020) 604–609.

79. Ha D, Schmidhuber J. World models. *arXiv preprint arXiv:1803.10122* (2018).

80. Hafner D, Lillicrap T, Ba J, Norouzi M. Dream to control: Learning behaviors by latent imagination. *arXiv preprint arXiv:1912.01603* (2019).

81. Kaiser L, Babaeizadeh M, Milos P, Osinski B, Campbell RH, Czechowski K, et al. Model-based reinforcement learning for atari. *arXiv preprint arXiv:1903.00374* (2019).

82. Janner M, Fu J, Zhang M, Levine S. When to trust your model: Model-based policy optimization. *Advances in neural information processing systems* **32** (2019).

83. Ha D, Schmidhuber J. Recurrent world models facilitate policy evolution. *Advances in neural information processing systems* **31** (2018).

84. Hussein A, Gaber MM, Elyan E, Jayne C. Imitation learning: A survey of learning methods. *ACM Computing Surveys (CSUR)* **50** (2017) 1–35.

85. Pomerleau DA. Efficient training of artificial neural networks for autonomous navigation. *Neural computation* **3** (1991) 88–97.

86. Ross S, Bagnell D. Efficient reductions for imitation learning. *Proceedings of the thirteenth international conference on artificial intelligence and statistics* (JMLR Workshop and Conference Proceedings) (2010), 661–668.

87. Arora S, Doshi P. A survey of inverse reinforcement learning: Challenges, methods and progress. *Artificial Intelligence* **297** (2021) 103500.

88. Ho J, Ermon S. Generative adversarial imitation learning. *Advances in neural information processing systems* **29** (2016).

89. Bojarski M. End to end learning for self-driving cars. *arXiv preprint arXiv:1604.07316* (2016).

90. Haykin S. *Neural networks: a comprehensive foundation* (: Prentice Hall PTR) (1998).

91. Rumelhart DE, Hinton GE, Williams RJ. Learning representations by back-propagating errors. *nature* **323** (1986) 533–536.

92. Salehinejad H, Sankar S, Barfett J, Colak E, Valaee S. Recent advances in recurrent neural networks. *arXiv preprint arXiv:1801.01078* (2017).

93. Graves A, Graves A. Long short-term memory. *Supervised sequence labelling with recurrent neural networks* (2012) 37–45.

94. Chung J, Gulcehre C, Cho K, Bengio Y. Empirical evaluation of gated recurrent neural networks on sequence modeling. *arXiv preprint arXiv:1412.3555* (2014).

95. Kipf TN, Welling M. Semi-supervised classification with graph convolutional networks. *arXiv preprint arXiv:1609.02907* (2016).

96. Hamilton W, Ying Z, Leskovec J. Inductive representation learning on large graphs. *Advances in neural information processing systems* **30** (2017).

97. Veličković P, Cucurull G, Casanova A, Romero A, Lio P, Bengio Y. Graph attention networks. *arXiv preprint arXiv:1710.10903* (2017).

98. Vaswani A, Shazeer N, Parmar N, Uszkoreit J, Jones L, Gomez AN, et al. Attention is all you need. *Advances in neural information processing systems* **30** (2017).

99. Lin T, Wang Y, Liu X, Qiu X. A survey of transformers. *AI open* **3** (2022) 111–132.

100. Lin Z, Feng M, Santos CNd, Yu M, Xiang B, Zhou B, et al. A structured self-attentive sentence embedding. *arXiv preprint arXiv:1703.03130* (2017).

101. Yu C, Ma X, Ren J, Zhao H, Yi S. Spatio-temporal graph transformer networks for pedestrian trajectory prediction. *Computer Vision–ECCV 2020: 16th European Conference, Glasgow, UK, August 23–28, 2020, Proceedings, Part XII 16* (Springer) (2020), 507–523.

102. Dugas D, Nieto J, Siegwart R, Chung JJ. Navrep: Unsupervised representations for reinforcement learning of robot navigation in dynamic human environments. *2021 IEEE international conference on robotics and automation (ICRA)* (IEEE) (2021), 7829–7835.

103. Pfeiffer M, Schaeuble M, Nieto J, Siegwart R, Cadena C. From perception to decision: A data-driven approach to end-to-end motion planning for autonomous ground robots. *2017 ieee international conference on robotics and automation (icra)* (IEEE) (2017), 1527–1533.

104. Hoeller D, Wellhausen L, Farshidian F, Hutter M. Learning a state representation and navigation in cluttered and dynamic environments. *IEEE Robotics and Automation Letters* **6** (2021) 5081–5088.

105 .Wang Y, He H, Sun C. Learning to navigate through complex dynamic environment with modular deep reinforcement learning. *IEEE Transactions on Games* **10** (2018) 400–412.

106 .Sun L, Zhai J, Qin W. Crowd navigation in an unknown and dynamic environment based on deep reinforcement learning. *IEEE Access* **7** (2019) 109544–109554.

107 .Lowe R, Wu YI, Tamar A, Harb J, Pieter Abbeel O, Mordatch I. Multi-agent actor-critic for mixed cooperative-competitive environments. *Advances in neural information processing systems* **30** (2017).

108 .Chen YF, Liu M, Everett M, How JP. Decentralized non-communicating multiagent collision avoidance with deep reinforcement learning. *2017 IEEE international conference on robotics and automation (ICRA)* (IEEE) (2017), 285–292.

109 .Matsuzaki S, Aonuma S, Hasegawa Y. Dynamic window approach with human imitating collision avoidance. *2021 IEEE International Conference on Robotics and Automation (ICRA)* (IEEE) (2021), 8180–8186.

110 .Chen C, Liu Y, Kreiss S, Alahi A. Crowd-robot interaction: Crowd-aware robot navigation with attention-based deep reinforcement learning. *2019 international conference on robotics and automation (ICRA)* (IEEE) (2019), 6015–6022.

111 .Stratton A, Hauser K, Mavrogiannis C. Characterizing the complexity of social robot navigation scenarios. *arXiv preprint arXiv:2405.11410* (2024).

112 .Yang S, Li T, Gong X, Peng B, Hu J. A review on crowd simulation and modeling. *Graphical Models* **111** (2020) 101081.

113 .Duives DC, Daamen W, Hoogendoorn SP. State-of-the-art crowd motion simulation models. *Transportation research part C: emerging technologies* **37** (2013) 193–209.

114 .Ijaz K, Sohail S, Hashish S. A survey of latest approaches for crowd simulation and modeling using hybrid techniques. *17th UKSIMAMSS international conference on modelling and simulation* (2015), 111–116.

115 .Helbing D, Molnar P. Social force model for pedestrian dynamics. *Physical review E* **51** (1995) 4282.

116 .Helbing D, Farkas I, Vicsek T. Simulating dynamical features of escape panic. *Nature* **407** (2000) 487–490.

117 .Golas A, Narain R, Lin M. Hybrid long-range collision avoidance for crowd simulation. *Proceedings of the ACM SIGGRAPH symposium on interactive 3D graphics and games* (2013), 29–36.

118 .Moussaïd M, Helbing D, Theraulaz G. How simple rules determine pedestrian behavior and crowd disasters. *Proceedings of the National Academy of Sciences* **108** (2011) 6884–6888.

119 .Xu S, Duh HBL. A simulation of bonding effects and their impacts on pedestrian dynamics. *IEEE Transactions on Intelligent Transportation Systems* **11** (2009) 153–161.

120 .Heliövaara S, Korhonen T, Hostikka S, Ehtamo H. Counterflow model for agent-based simulation of crowd dynamics. *Building and Environment* **48** (2012) 89–100.

121 .Guo RY. Simulation of spatial and temporal separation of pedestrian counter flow through a bottleneck. *Physica A: Statistical Mechanics and its Applications* **415** (2014) 428–439.

122 .Tordeux A, Chraibi M, Seyfried A. Collision-free speed model for pedestrian dynamics. *Traffic and Granular Flow'15* (Springer) (2016), 225–232.

123 .Curtis S, Manocha D. Pedestrian simulation using geometric reasoning in velocity space. *Pedestrian and evacuation dynamics 2012* (Springer) (2014), 875–890.

124 .Wolinski D, J Guy S, Olivier AH, Lin M, Manocha D, Pettré J. Parameter estimation and comparative evaluation of crowd simulations. *Computer Graphics Forum* (Wiley Online Library) (2014), vol. 33, 303–312.

125 .Berseth G, Kapadia M, Haworth B, Faloutsos P. Steerfit: Automated parameter fitting for steering algorithms. *Simulating Heterogeneous Crowds with Interactive Behaviors* (: AK Peters/CRC Press) (2016), 229–246.

126 .Kreiss S. Deep social force. *arXiv preprint arXiv:2109.12081* (2021).

127 .Hoogendoorn S, HL Bovy P. Simulation of pedestrian flows by optimal control and differential games. *Optimal control applications and methods* **24** (2003) 153–172.

128 .Guy SJ, Chhugani J, Kim C, Satish N, Lin M, Manocha D, et al. Clearpath: highly parallel collision avoidance for multi-agent simulation. *Proceedings of the 2009 ACM SIGGRAPH/Eurographics Symposium on Computer Animation* (2009), 177–187.

129 .Karamouzas I, Sohre N, Narain R, Guy SJ. Implicit crowds: Optimization integrator for robust crowd simulation. *ACM Transactions on Graphics (TOG)* **36** (2017) 1–13.

130 .Guy SJ, Chhugani J, Curtis S, Dubey P, Lin MC, Manocha D. Pledestrians: A least-effort approach to crowd simulation. *Symposium on computer animation* (2010), 119–128.

131 .Hoogendoorn SP, Bovy PH. Pedestrian route-choice and activity scheduling theory and

models. *Transportation Research Part B: Methodological* **38** (2004) 169–190.

132 .Seitz MJ, Köster G. Natural discretization of pedestrian movement in continuous space. *Physical Review E—Statistical, Nonlinear, and Soft Matter Physics* **86** (2012) 046108.

133 .Howard A. The robotics data set repository (radish). *http://radish. sourceforge. net/* (2003).

134 .Dobrevski M, Skočaj D. Adaptive dynamic window approach for local navigation. *2020 IEEE/RSJ International Conference on Intelligent Robots and Systems (IROS)* (IEEE) (2020), 6930–6936.

135 .Xia F, Zamir AR, He Z, Sax A, Malik J, Savarese S. Gibson env: Real-world perception for embodied agents. *Proceedings of the IEEE conference on computer vision and pattern recognition* (2018), 9068–9079.

136 .Chang A, Dai A, Funkhouser T, Halber M, Niessner M, Savva M, et al. Matterport3d: Learning from rgb-d data in indoor environments. *arXiv preprint arXiv:1709.06158* (2017).

137 .Dugas D, Nieto J, Siegwart R, Chung JJ. Ian: Multi-behavior navigation planning for robots in real, crowded environments. *2020 IEEE/RSJ International Conference on Intelligent Robots and Systems (IROS)* (IEEE) (2020), 11368–11375.

138 .Corbetta A, Bruno L, Muntean A, Toschi F. High statistics measurements of pedestrian dynamics. *Transportation Research Procedia* **2** (2014) 96–104.

139 .Lerner A, Chrysanthou Y, Lischinski D. Crowds by example. *Computer graphics forum* (Wiley Online Library) (2007), vol. 26, 655–664.

140 .Gao Y, Huang CM. Evaluation of socially-aware robot navigation. *Frontiers in Robotics and AI* **8** (2022) 721317.

141 .Francis A, Pérez-d'Arpino C, Li C, Xia F, Alahi A, Alami R, et al. Principles and guidelines for evaluating social robot navigation algorithms. *arXiv preprint arXiv:2306.16740* (2023).

142 .Singamaneni PT, Favier A, Alami R. Watch out! there may be a human. addressing invisible humans in social navigation. *2022 IEEE/RSJ International Conference on Intelligent Robots and Systems (IROS)* (IEEE) (2022), 11344–11351.

143 .Pirk S, Lee E, Xiao X, Takayama L, Francis A, Toshev A. A protocol for validating social navigation policies. *arXiv preprint arXiv:2204.05443* (2022).

144 .Pellegrini S, Ess A, Schindler K, Van Gool L. You'll never walk alone: Modeling social behavior for multi-target tracking. *2009 IEEE 12th international conference on computer vision* (IEEE) (2009), 261–268.

145 .[Dataset] Makoviichuk D, Makoviychuk V. rl-games: A high-performance framework for reinforcement learning. `https://github.com/Denys88/rl_games` (2021).

146 .Akiba T, Sano S, Yanase T, Ohta T, Koyama M. Optuna: A next-generation hyperparameter optimization framework. *Proceedings of the 25th ACM SIGKDD international conference on knowledge discovery & data mining* (2019), 2623–2631.

147 .[Dataset] Stüvel SA. Python-rvo2 library. `https://github.com/sybrenstuvel/Python-RVO2` (2025).

148 .[Dataset] Gao Y. Pysocialforce. `https://github.com/yuxiang-gao/PySocialForce` (2025).

149 .Sakai A, Ingram D, Dinius J, Chawla K, Raffin A, Paques A. Pythonrobotics: a python code collection of robotics algorithms. *arXiv preprint arXiv:1808.10703* (2018).

150 .Gleave A, Taufeeque M, Rocamonde J, Jenner E, Wang SH, Toyer S, et al. imitation: Clean imitation learning implementations. *arXiv preprint arXiv:2211.11972* (2022).

151 .Everett M, Chen YF, How JP. Motion planning among dynamic, decision-making agents with deep reinforcement learning. *2018 IEEE/RSJ International Conference on Intelligent Robots and Systems (IROS)* (IEEE) (2018), 3052–3059.

152 .Chen C, Hu S, Nikdel P, Mori G, Savva M. Relational graph learning for crowd navigation. *2020 IEEE/RSJ International Conference on Intelligent Robots and Systems (IROS)* (IEEE) (2020), 10007–10013.

153 .Linh K, Cox J, Buiyan T, Lambrecht J, et al. All-in-one: A drl-based control switch combining state-of-the-art navigation planners. *2022 International Conference on Robotics and Automation (ICRA)* (IEEE) (2022), 2861–2867.

154 .Redmon J. Yolov3: An incremental improvement. *arXiv preprint arXiv:1804.02767* (2018).

155 .Bochkovskiy A, Wang CY, Liao HYM. Yolov4: Optimal speed and accuracy of object detection. *arXiv preprint arXiv:2004.10934* (2020).

156 .Bewley A, Ge Z, Ott L, Ramos F, Upcroft B. Simple online and realtime tracking. *2016 IEEE international conference on image processing (ICIP)* (IEEE) (2016), 3464–3468.

157 .Wojke N, Bewley A, Paulus D. Simple online and realtime tracking with a deep association metric. *2017 IEEE international conference on image processing (ICIP)* (IEEE) (2017), 3645–3649.
